# Synthesis of Functional Water-Soluble Polyesters Based on Citric Acid and Dimethylolpropionic Acid

**DOI:** 10.1021/acspolymersau.5c00196

**Published:** 2026-02-20

**Authors:** Anna Kruglhuber, Clemens Bernhard, Susanne Boye, Markus Wierer, Albena Lederer, Clemens Schwarzinger, Klara M. Saller

**Affiliations:** † 27266 Institute for Chemical Technology of Organic Materials, Johannes Kepler University Linz, Altenbergerstrasse 69, 4020 Linz, Austria; ‡ 28408 Leibniz-Institut für Polymerforschung Dresden, Department Advanced Macromolecular Structure Analysis, Hohe Strasse 6, 01069 Dresden, Germany; § Institute for Analytical Chemistry, Johannes Kepler University Linz, Altenbergerstrasse 69, 4020 Linz, Austria; ∥ Stellenbosch University, Department Chemistry and Polymer Science, Private Bag X1, Matieland 7602, South Africa

**Keywords:** functional polyester, water-soluble polyester, charged polyester, citric acid, biobased monomers, sulfonation

## Abstract

Aiming for renewable polymers with potential recyclability and biodegradability, polyesters are very promising due to an increasing number of available biobased monomers and ester bonds that can be hydrolyzed under specific conditions. Citric acid, for example, is a biobased, nontoxic, cheap, and easily available resource. Its multifunctionality enables the synthesis of polyesters with free carboxy groups, thus providing possibilities for further functionalization and cross-linking. Citric acid and dimethylolpropionic acid were used to synthesize water-soluble polyesters via melt polycondensation at 150 °C without the need for potentially hazardous catalysts. The resulting polyesters displayed a significant amount of free carboxylic acid groups (8 mmol g_polyester_^–1^), and reasonable number-average molar masses up to 5200 g mol^–1^. Via postsynthetical (partial) neutralization procedures using KOH, charged moieties could be successfully incorporated to further increase hydrophilicity. The degree of neutralization proved to be well controllable. This enables tunability of the final properties, as remaining free carboxy groups can be used for further modifications. Alternative to carboxylate moieties, the introduction of sulfonate groups promotes hydrophilicity. For this purpose, unsaturated polyesters were synthesized that contained varying amounts of maleic anhydride as a third monomer. Postsynthetical sulfonation was performed via the Michael addition of sodium sulfite, introducing a significant number of sulfonate groups. Neutralization and sulfonation were performed in aqueous solution, leading to slight decreases in molar mass due to hydrolysis. The extent of the reduction was successfully reduced by optimizing both procedures. The synthesized water-soluble polyesters carry a substantial number of functional groups. They have high potential as precondensates for cross-linked, water-absorbing materials to be used in agriculture and biomedicine, for which biodegradability is a crucial property.

## Graphical abstract

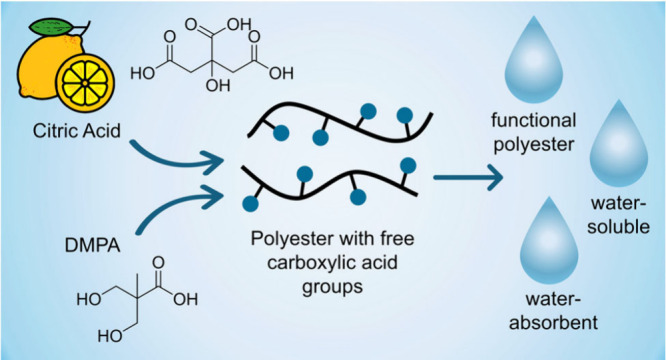

## 1 Introduction

The development of sustainable alternatives to conventional polymeric materials based on petrochemicals is urgent, considering the heap of environmental problems that need to be tackled.^1–4^ The substitution of traditional monomers to biobased and renewable educts hereby is a well-established strategy.^1,4,5^ When further aiming for potential biodegradability and recyclability, polyesters are especially attractive, as ester bonds are hydrolyzable when exposed to specific conditions.^2,3,6–8^ Functional polyesters are specialized polymeric materials that can be tuned to display specific properties via incorporation of functional groups and reactive binding sites.^7–10^

The utilization of already functionalized monomers is one option to synthesize functional polyesters.^6–8^ Due to issues such as side reactions^6,9,11^ and gelation,^9^ protection and deprotection steps are usually required,^6,9,11^ thus making the overall process more complicated and ecologically problematic.^3,12^ Postsynthetical functionalization is a comparatively more simplistic processing route. Respective reactions such as neutralization or sulfonation procedures are usually well controllable, thus enabling fine-tuning of the number of functionalized positions.^10^ To conduct postsynthetical functionalization, the polyester precondensates have to display reactive sites or functional moieties that can be modified. Due to its multifunctionality, citric acid (CA) is a promising monomer in this respect.^1^

Citric acid is a natural tricarboxylic acid^13,14^ that is characterized by its accessibility and overall safety of usage.^13^ While both the primary and tertiary COOH groups take part in (poly)­condensation reactions, the esterification of primary carboxy groups is preferred.^1^ This allows the synthesis of polyester structures with a significant number of free acidic functional groups that can be utilized for subsequent modification.^7,15^ The hydroxy group of citric acid is reported to be unreactive.^1^ Given its many application areas, citric acid production already takes place on an industrial scale via biosynthetic fermentation processes, making it an inexpensive monomer as well.^14^ For fermentation, mostly the black mold strain *Aspergillus niger* is employed, while molasses is used as the required sugar source,^16^ making citric acid a highly sustainable resource.^1^

Incorporation of charged moieties is a way to functionalize polyester chains by increasing their hydrophilicity. When citric acid is used as the monomer,^1,17^ polyester chains display a significant number of free acidic functional groups^1^ that can be easily deprotonated via postsynthetical treatment with a strong base,^7,15^ although a decrease in molar mass needs to be considered due to the sensitivity of the polyesters toward hydrolysis at harsh pH conditions.^7,9^ By additionally selecting an alcohol component such as dimethylolpropionic acid (DMPA), overall functionality of the polyester system can be enhanced even more, as a higher number of free carboxy groups are incorporated.^3,18^

Apart from carboxylate groups, other charged functional groups such as sulfonate moieties can also be used to increase hydrophilicity.^7,10,19,20^ Many different routes for the incorporation of sulfonate groups are reported in the literature,^15,21–23^ most of them utilizing harsh chemicals such as oleum or sulfuric acid, thus leading to a variety of environmental, toxicological, and technical problems.^23^ A more environmentally friendly sulfonation method is the postsynthetical Michael addition of sulfonate groups to double bonds,^19,23,24^ as reagents such as sodium sulfite (Na_2_SO_3_) can be used^19^ at mild reaction conditions and in aqueous medium. Other advantages include the high efficiency, irreversible character, and the fact that no catalyst is required.^25^ To perform Michael addition reactions, synthesis of unsaturated polyester precondensates is necessary using comonomers like maleic anhydride (MAn), maleic acid,^26^ or itaconic acid.^27,28^ A side reaction that has been reported with respect to unsaturated polyesters is the Ordelt reaction, wherein a hydroxy group undergoes addition to an available double bond, thus potentially leading to cross-linking and branching during polycondensation.^4^

Citric acid-based polyesters have a variety of potential application areas, most of them being based on hydrophilicity. Hereby, the market for superabsorbent polymers (SAPs) represents an important sector, ranging from hygiene^29–31^ to agricultural applications.^32,33^ In agriculture, SAPs can be used to retain moisture within the soil, thereby improving water uptake of plants during dry periods.^34^ Additionally, essential macronutrients can be incorporated,^34,35^ which are thus prevented from quick dissolution and available to plants for prolonged timespans. As the SAPs are dispersed freely within the soil, it is crucial to ensure the nontoxicity and biodegradability of the used materials.^34^ Citric acid-based polyesters are relevant for biomedical applications as well,^36–38^ including for example drug delivery and wound care.^36,37^ Contrary to currently used materials such as polylactic acid^6,38^ or polycaprolactone,^6^ tunability of properties such as degradation rate, durability, and elasticity is possible via the available free functional groups.^36–38^ Additionally, hydrophilic materials display improved cell adhesion, which is important for efficient interaction.^39^

In our study, we report the first synthesis of polyesters using the combination of the two multifunctional monomers, citric acid and dimethylolpropionic acid, leading to a very high number of carboxylic acid groups along the polyester chain. Due to this structural resemblance to polyacrylic acid (PAA), similar modifications could be applied while ester bonds promote biodegradability and allow recyclability in contrast to the very stable C–C backbone in PAA. Water-absorbing properties of PAA are introduced by neutralization to carboxylates to provide charged moieties with increased hydrophilicity and cross-linking of the remaining carboxy groups to form a water-insoluble network. We designed our water-soluble polyester systems with charged functionalities and varying concentrations of carboxylic acid groups to fulfill exactly these conditions. First, the synthesis and (partial) neutralization of citric acid–dimethylolpropionic acid polyesters was investigated. Further, we used the versatility of polyester chemistry to also incorporate sulfonate groups via Michael addition onto maleic acid building blocks. Both polyester syntheses via melt polycondensation and postmodification via neutralization or sulfonation posed challenges like gelation and reduced molar masses needing careful consideration and optimization of reaction parameters. These specialized water-soluble polyesters carry great potential as precondensates for mainly biobased, likely biodegradable and recyclable materials with fine-tuned water-absorbing properties for agricultural and biomedical applications.

## 2 Experimental Section

### 2.1 Monomers

Citric acid (CA; Sigma-Aldrich, 99%), maleic anhydride (MAn; Alfa Aesar, 98+%), and 2,2-bis­(hydroxy­methyl)­prop­ionic acid (dimethylolpropionic acid, DMPA; Sigma-Aldrich, 98%) were utilized as monomers within the performed polycondensation reactions. The monomers were used without further purification.

### 2.2 Polymer Synthesis

Polyester synthesis was conducted with a slight acid surplus with the molar ratio between primary carboxy and hydroxy functional groups of the utilized monomers defined as 1.05:1. Syntheses were scaled to yield 150 or 300 g of polyester product using either 250 or 500 mL flat flange glass reactors with four-neck lids. The individual setups were equipped with a mechanical stirrer and a nitrogen inlet and were connected to a vacuum system with pressure control. The fourth neck was used for monomer addition and sample extraction. The reaction mixture was electrically heated by using a heating mantle. Temperature control was conducted with a temperature sensor positioned between the heating mantle and the reactor wall. The temperature of the reaction mass was set to 150 °C and monitored with a separate sensor. PES-01 was synthesized using cyclopentyl methyl ether as an entrainer for promoting water removal via azeotropic distillation. In this case, a Dean–Stark apparatus was built in the reaction apparatus to collect and monitor the reaction water. The polycondensation reaction in further syntheses was promoted by reducing the pressure for water removal. While this setup prohibited an accurate monitoring of reaction water, the application of an additional chemical could be avoided.

For synthesis, the acid components (CA, MAn) were loaded into the reactor and melted by raising the temperature to 150 °C in a continuous N_2_ flow (50–100 mL min^–1^). A slow increase of temperature was employed so that overheating of the mixture and beginning degradation of CA could be prevented. If only CA was used, melting took approximately 30 or 60 min for 150 and 300 g, respectively. This step was accelerated with an increasing amount of maleic anhydride due to its lower melting point. After obtaining a clear melt of the acid components, we DMPA added in four equal portions, waiting for the clearance point before each subsequent addition. The total DMPA addition step took between 20 and 25 min. The point at which all educts were added and gave a clear melt within the reactor at 150 °C was set as starting time *t*_0_. Once *t*_0_ was reached, a stepwise decrease in pressure was employed until 50–100 mbar was reached. For some MAn-CA-DMPA polyesters (PES-08, PES-10, PES-12), the decrease only took place after 1 h of reaction time at atmospheric pressure under a nitrogen stream. The end point of the reaction was reached once the viscosity of the reaction mass increased significantly. The polyester product was transferred into an aluminum dish and left to cool.

#### 2.2.1 Neutralization Procedure

Postsynthetical neutralization of CA-DMPA polyester precondensates (Scheme 1) was performed with an aqueous potassium hydroxide solution (ca. 4 mol L^–1^). The titer of the solution was determined via titration against potassium hydrogen phthalate (VWR, ≥99.5%). Subsequently, KOH solution was added gravimetrically, where different neutralization degrees (25, 50, 75, or 100%) were aimed for. For optimization purposes, the solvent (water/acetone/no solvent) as well as the concentration of the polyester precondensate within the solvent were varied. The reaction time was set at 1 h. Subsequently, the solvent was removed via rotary evaporation. The samples were dried for 3–4 h in a vacuum oven (70 °C) before SEC analysis and acid value measurements were conducted. Neutralization degrees were calculated based on acid values. Experiments were performed on both small (5 g of precondensate) and large scales (50 g of precondensate).

**Scheme 1:**
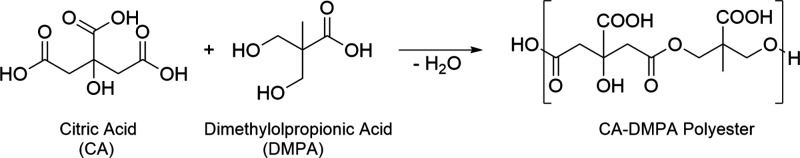
Synthesis of CA-DMPA Polyester Precondensate from Citric Acid and Dimethylolpropionic Acid, Followed by Neutralization with a Strong Base^a^

a Due to simplification, only reaction of the primary carboxylic acid groups of CA is indicated, while the tertiary COOH groups also participate in the reaction to a certain extent.

#### 2.2.2 Sulfonation Procedure

MAn-CA-DMPA polyesters were functionalized by sulfonating unsaturated positions via the Michael addition of sodium sulfite (Na_2_SO_3_; Alfa Aesar, anhydrous, 98%), as shown in Scheme 2. Using quantitative ^1^H NMR, the amount of double bonds within the precondensate was determined, and the Na_2_SO_3_ was added in a molar ratio of 1:1. To prevent harsh pH conditions, the Na_2_SO_3_ solution was neutralized with HCl prior to addition. For optimization, different concentrations of precondensate in aqueous solution (9–29 wt %) and several temperature conditions (25–70 °C, control via oil bath) were tested. Reactions were performed for 24 h under an argon atmosphere. The product was transferred to an aluminum dish to let the majority of water evaporate overnight. Subsequently, the sulfopolyesters were dried for 3–4 h in a vacuum oven (70 °C) before analysis was conducted. The residual amount of double bonds was used to calculate the degree of sulfonation. Experiments were performed on both small (4 g of precondensate) and large scales (40 g of precondensate).

**Scheme 2:**
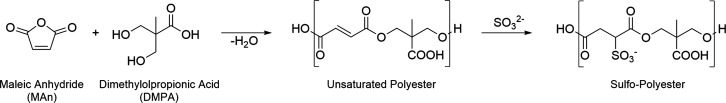
Synthesis of Unsaturated Polyester Precondensate from Maleic Anhydride and Dimethylolpropionic Acid, Followed by Sulfonation via Michael Addition of a Sulfite

### 2.3 Polymer Analysis

#### 2.3.1 NMR Spectroscopy

^1^H NMR spectra were recorded on a Bruker Avance 300 MHz instrument using either deuterated dimethyl sulfoxide (DMSO-*d*_6_; Deutero 99.8%) or deuterium oxide (D_2_O; Deutero 99.9%) as the solvent. Solutions were prepared at a concentration of approximately 25 mg mL^–1^. Peaks are given in parts per million relative to tetramethylsilane. For further investigation and peak assignment, selected samples were examined via ^13^C­(-APT) measurements and/or 2D NMR spectroscopy including COSY, HSQC, and HMBC experiments. Representative NMR spectra are shown in the Supporting Information (SI).

The OH conversion of DMPA was determined based on its methyl peaks (SI, Figures S1 and S8), where the respective peak areas (*A*) for monomers, monoesters, and diesters were used. For CA-DMPA polyesters, eq 1 (corresponding to measurement in D_2_O) was used, while for MAn-CA-DMPA polyesters, eq 2 (corresponding to measurement in DMSO-*d*_6_) was used. As sulfopolyester signals displayed a significant downfield shift with an increasing degree of sulfonation, the integrals from eq 2 were individually adjusted.

For quantitative ^1^H NMR measurements, benzoic acid (Merck, titrimetric standard) was used as the internal standard in DMSO-*d*_6_. Due to differences in solubility, NMR spectra of sulfopolyesters were measured in D_2_O using potassium hydrogen phthalate as an internal standard. For calculation, the integrals of the double bond region (7.00–6.20 ppm, DMSO-*d*_6_/D_2_O), benzoic acid (8.05–7.56 ppm, 3H, DMSO-*d*_6_), and potassium hydrogen phthalate (7.90–7.50 ppm, 4H, D_2_O) were used (more details in the SI, Section 1.4).

1 $$\mathrm{OH conversion}/\%=\frac{A\left(1.32\text{−}1.23\;\mathrm{ppm}\right)+\frac{A\left(1.23\text{−}1.17\;\mathrm{ppm}\right)}{2}}{A\left(1.32\text{−}1.23\;\mathrm{ppm}\right)+A\left(1.23\text{−}1.17\;\mathrm{ppm}\right)+A\left(1.17\text{−}1.14\;\mathrm{ppm}\right)}$$

2 $$\mathrm{OH conversion}/\%=\frac{A\left(1.28\text{−}1.08\;\mathrm{ppm}\right)+\frac{A\left(1.08\text{−}1.01\;\mathrm{ppm}\right)}{2}}{A\left(1.28\text{−}1.08\;\mathrm{ppm}\right)+A\left(1.08\text{−}1.01\;\mathrm{ppm}\right)+A\left(1.01\text{−}0.97\;\mathrm{ppm}\right)}$$

#### 2.3.2 SEC

Size exclusion chromatography (SEC) in an aqueous medium was performed with an Agilent 1260 pump system and diode array detector connected to a Wyatt Optilab T-rEX refractive index detector. Selected measurements were repeated including a Wyatt DAWN HELEOS-II multiangle light scattering (MALS) and a Wyatt ViscoStar Neon detector. A neutral aqueous buffer containing 0.1 M NaNO_3_ (Sigma-Aldrich, 99%) and 0.02 wt % NaN_3_ (Alfa Aesar, 99%) in Milli-Q water (purified using a Milli-Q-Advantage A10 system, Merck Millipore) was used as the solvent at a flow rate of 1 mL min^–1^. Analyte solutions in buffer (2–3 mg mL^–1^) were separated at 25 °C using an Agilent PL aquagel-OH 20 column (5 μm, 300 × 7.5 mm, 100–20 000 Da). Relative molar masses for all samples were calculated based on calibration with pullulan standards (22 000–342 g mol^–1^) using the refractive index detector. Representative samples were additionally measured and interpreted using the viscosity detector for determining the viscometric, hydrodynamic radius *R*_h,v_ and intrinsic viscosity [η] as well as the MALS detector for absolute molar mass determination including fitting of the results (SI, Figure S11).

An Agilent 1200 system including a diode array detector and an external PerkinElmer 200 refractive index detector was used for SEC analysis in tetrahydrofuran (unstabilized, ≥99.9%, Carl Roth). Samples were separated at 40 °C with a Phenogel precolumn (30 × 4.6 mm, 5 μm, linear) and three Phenogel columns (300 × 4.6 mm, 5 μm) with pore sizes of 50, 500, and 10 000 Å. Sample concentrations of ca. 2 mg mL^–1^, an injection volume of 50 μL, and a flow rate of 0.35 mL min^–1^ were chosen for analysis. Molar masses were calculated as polystyrene equivalents using the refractive index detector and by choosing the elution volume of the smallest PS standard (162 g mol^–1^) as the cutoff.

#### 2.3.3 HPLC-MS

HPLC-MS measurements were performed on an Agilent Ion Mobility LC/MS Q-TOF (G6560A) to analyze small oligoester species of representative samples. Samples were dissolved in Milli-Q water (purified using a Milli-Q EQ 7000, Merck Millipore) at a concentration of 1 mg mL^–1^. 5 μL of sample solution was injected and separated with a Phenomenex Kinetex C18 column (2.6 μm, 50 × 3 mm) at a flow rate of 0.5 mL min^–1^ using a water/acetonitrile gradient (SI, Table S2). The analytes were ionized in a Dual AJS ESI source and separated in an *m*/*z* range of 100–1700 in positive mode. The drying/sheath gas temperature was set at 350 °C. The drying gas flow rate was 10.5 L min^–1^, and the sheath gas flow rate was 11 L min^–1^ (both nitrogen). The nebulizer pressure was 40 psi, the capillary voltage 3500 V, the nozzle voltage 1000 V, and the fragmentor voltage 400 V.

#### 2.3.4 MALDI Mass Spectrometry

Matrix-assisted laser desorption/ionization time-of-flight mass spectra were recorded in a mass range of 400–4000 *m*/*z* on a Bruker UltrafleXtreme instrument in reflectron mode. Additional measurements were performed in linear mode to roughly estimate number- and weight-average molar masses. 2,5-Dihydroxybenzoic acid (DHB, Sigma-Aldrich, >99.0%) was used as the matrix, and sodium trifluoroacetate (Thermo Scientific, 98%) was used as the ionization agent. DHB and polyethylene glycol standards with molar masses of 600, 1500, and 3350 g mol^–1^ (Fluka) were dissolved in THF at concentrations of 10 mg mL^–1^, while polyester samples were dissolved at 10 mg mL^–1^ in Milli-Q H_2_O. For the ionization agent, a concentration of 1 mg mL^–1^ in THF was chosen. Matrix, sample/standard, and salt solutions were mixed in a 100:10:1 ratio and spotted onto the MALDI target via the dried droplet method. Spectra were processed using the Bruker FlexAnalysis Software 3.0, and molar masses were calculated in Bruker PolyTools 2.0. Interpretations were assisted by the in-house developed MALDI interpretation tool *MALINTO*.^40^

#### 2.3.5 DSC

A Mettler Toledo DSC 3+ differential scanning calorimeter was used to investigate the glass-transition temperatures (*T*_g_). A sample size of approximately 3 mg was weighed into 40 μL aluminum crucibles. Samples were first heated to 80 °C and then cooled to −80 °C. The second heating curve to 200 °C was evaluated. Heating and cooling rates of 20 K min^–1^ were used.

#### 2.3.6 Acid Value Determination

For determination of the acid value, 0.2–0.3 g of sample was dissolved in a small volume of deionized water. If necessary, the flask was placed in a water bath (40 °C) for 15 min to foster dissolution. Once the sample was cooled to room temperature again, titration with a 0.1 M ethanolic potassium hydroxide solution was performed, and the titer was determined via titration of potassium hydrogen phthalate. Phenolphthalein was used as the indicator. The acid value (AV/mg_KOH_ g^–1^) was calculated based on eq 3 and could be used for calculation of the COOH conversion as well as neutralization degrees.

3 $$\mathrm{AV}=\frac{n_{\mathrm{COOH}}M_{\mathrm{KOH}}}{m_{\mathrm{PES}}}$$

where

- AV = acid value/mg_KOH_ g^–1^
- *n*_COOH_ = amount of free carboxylic acid groups/mmol
- *M*_KOH_ = molar mass of KOH/g mol^–1^
- *m*_PES_ = mass of polyester/g

## 3 Results and Discussion

### 3.1 Synthesis with Citric Acid and Dimethylolpropionic Acid

Using citric acid (CA) and dimethylolpropionic acid (DMPA) as monomers leads to the synthesis of polyesters with a significant amount of free carboxy functional groups. They provide an opportunity for further functionalization, potentially catering to specialized applications such as biodegradable superabsorbent polymers (SAPs).^3,10,17^ Therefore, optimization of CA-DMPA precondensate synthesis represents the first important step toward biobased functional polyesters. The main investigations concerned the optional utilization of an entrainer for the removal of reaction water, efficient control of temperature and pressure, as well as the timing of monomer addition to the reactor. CA-DMPA syntheses with corresponding reaction parameters and results are listed in Table 1.

**Table 1:** Summary of the Performed Syntheses and Reaction Parameters, with Corresponding Results in the Form of OH Conversion (Based on NMR) and *M*_n_ and *M*_w_ Values (Based on Aqueous SEC-RI with Pullulan Calibration)

Sample	Batch size/g	Addition of monomers	H_2_O removal	OH conv. (NMR)/%	*M*_n_/g mol^–1^	*M*_w_/g mol^–1^
PES-01	150	simultaneous	entrainer	81	5900	9200
PES-02	150	simultaneous	100 mbar	86	7600	15100
PES-03	150	stepwise	100 mbar	84	7100	12700
PES-04	150	stepwise	100 mbar	82	5800	8900
PES-05	300	stepwise	100 mbar	83	6700	11900
PES-06	300	stepwise	100 mbar	89	10000^a^	49600^a^

a Size exclusion limit of used Agilent PL aquagel-OH 20 column exceeded.

In experiment PES-01, synthesis with an entrainer was tested, where 35 mL of cyclopentyl methyl ether (CPME) was used. Not only would this enable synthesis at atmospheric pressure and monitoring of the water formation, but due to the slight dilution of the reaction mixture, overall homogeneity would also be fostered while preventing premature gelation. The main difficulty was the effective removal of the entrainer from the reaction mixture. Residual CPME was detected in PES-01, resulting in a distinct ether smell. As this is undesirable for final products, syntheses without any entrainer are preferable. Instead, the pressure within the reactor was reduced to 100 mbar to remove reaction water, which worked well as reflected in the overall higher OH conversions of 82–86%. Even though the simultaneous addition of both monomers led to a good OH conversion of 86% (PES-02), significant homogeneity issues (agglomeration of monomer) were experienced without the presence of CPME. A stepwise addition (premelting of CA followed by the addition of DMPA in four portions) could avoid this behavior while additionally preventing the self-condensation of DMPA.^3,18,41^ HPLC (SI, Figure S13) and MALDI (Figure 1) measurements verified that the reactivity of the tertiary hydroxyl group of citric acid could be neglected, and self-condensation of DMPA only rarely occurred in these CA-DMPA polyester systems. Full MALDI mass spectra can be found in the SI (Figure S14).

**Figure 1:**
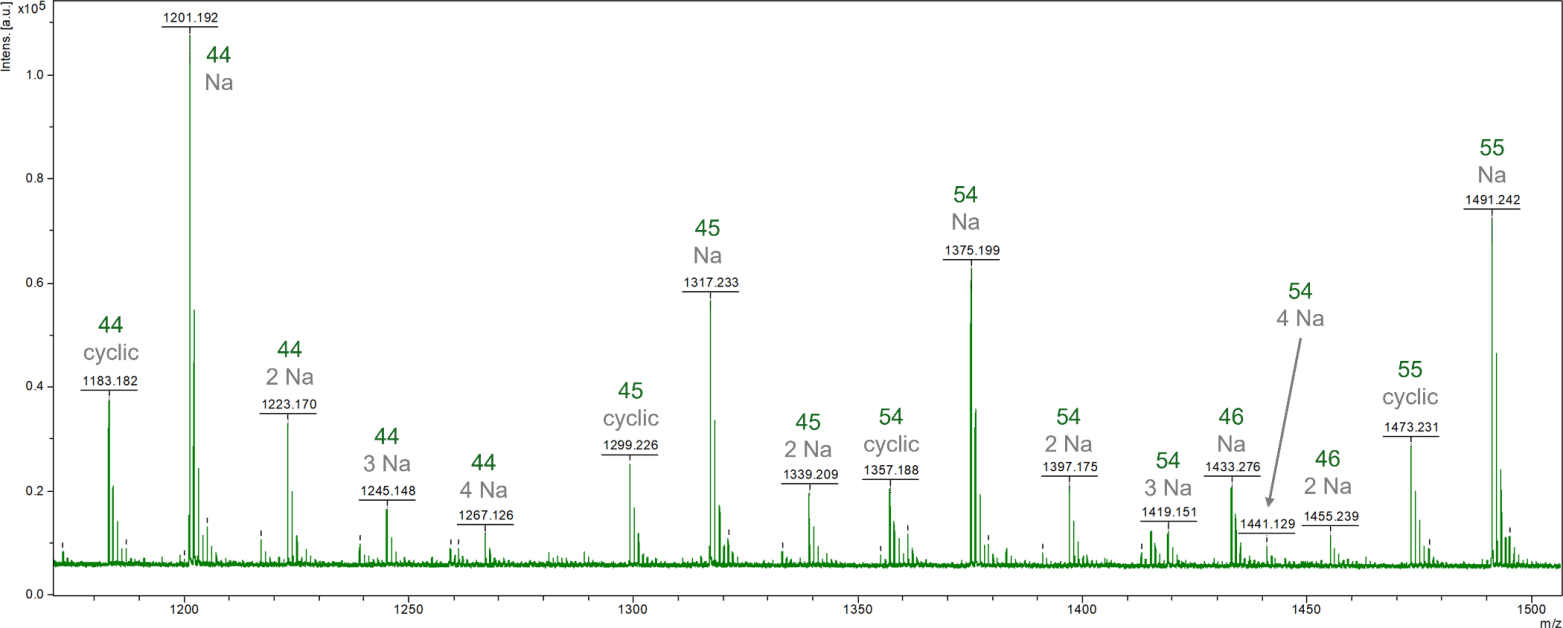
MALDI-TOF mass spectrum of a CA-DMPA polyester precondensate (PES-05). The peaks are labeled in green with their components according to a short nomenclature system, in which the first number corresponds to CA, while the second number corresponds to DMPA monomers. Na adducts and cyclic components are additionally indicated in gray.

Experiments performed under the described reaction conditions (PES-03, PES-04, PES-05) displayed reasonable reproducibility with OH conversions of 82–84% and *T*_g_ values of approximately 50 °C. The *T*_g_ hereby shows a dependence on the conversion, which was well observable for PES-02 (86%, 58 °C). Scale-up of the synthesis (PES-05) did not lead to major changes of the reaction procedure except a deviating reaction time, which seems to require careful adjustments for individual setups. All syntheses were performed at 150 °C. Due to the viscosity and bubble formation of the reaction mass, slight fluctuations in temperature were inevitable; 170 °C was defined as the maximum acceptable temperature to prevent degradation of CA.

The OH conversions were calculated based on the methyl region of DMPA in ^1^H NMR (Figure 2, details in the SI, Figure S1). The splitting of ester peaks was presumably caused by a reaction with either primary or tertiary carboxylic acid groups of CA. A more detailed peak assignment of CA-DMPA polyesters was performed with the help of ^13^C and 2D NMR measurements (SI, Figures S2–S4) and is in accordance with related literature.^1,3,42^

**Figure 2:**
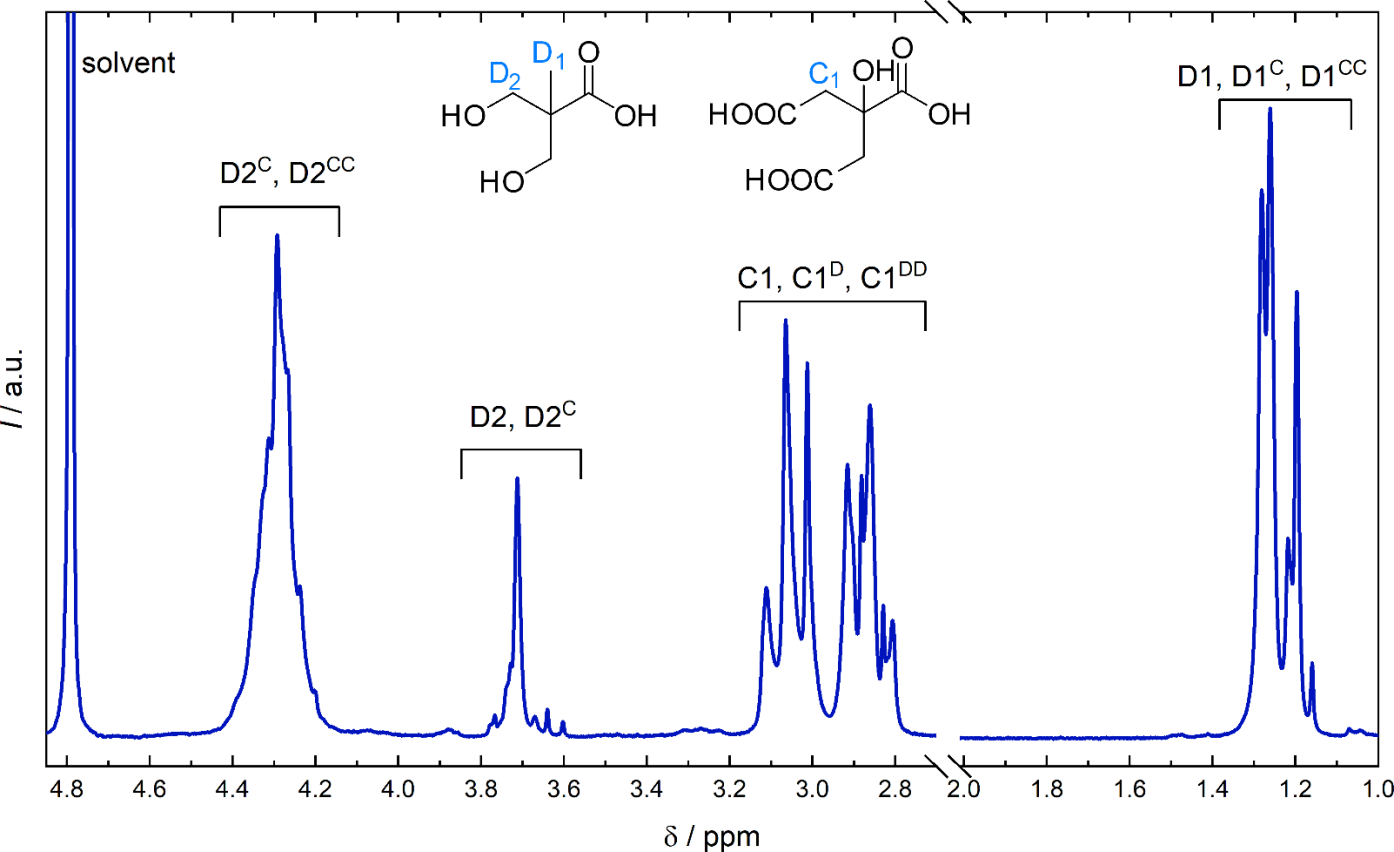
^1^H NMR spectrum of PES-05 (in D_2_O). The displayed structures (CA and DMPA) indicate the nomenclature system according to which the signals are assigned. Superscripts indicate reaction partner(s) within the polyester chain (“D” for DMPA, “C” for CA). They also specify whether the signal corresponds to a monomer (no superscript, e.g., D1), a monoester (e.g., D1^C^), or a diester (e.g., D1^CC^).

SEC analysis of CA-DMPA precondensates could be performed using both aqueous NaNO_3_ buffer and THF as the solvent, resulting in significant differences of the results as discussed in more detail in the SI (Section S2). Aqueous SEC results are discussed in this section for direct comparison with neutralized samples (Section 3.2). Calculating the molar mass for the monomers using the refractive index detector and pullulan calibration revealed a significant overestimation for citric acid (3650 instead of 192 g mol^–1^) while giving reasonable results for DMPA (148 instead of 134 g mol^–1^). Due to this result, an overestimation of molar masses for CA-DMPA precondensates was assumed and confirmed by absolute molar mass determination of selected samples using a multiangle light scattering detector as well as MALDI measurements in linear mode. For sample PES-03, RI gave an *M*_n_ value of 7100 g mol^–1^, while light scattering resulted in 2100 g mol^–1^. MALDI gave an estimated value of 1600 g mol^–1^. The corresponding full MALDI mass spectrum can be found in the SI (Figure S15).

Since oligoester regions are underrepresented in the MALS signal, a fitting was performed to significantly increase the mass accuracy (SI, Figure S11). Results from the viscosity detector were used to construct a Kuhn–Mark–​Houwink–Sakurada (KMHS) plot according to eq 4, which revealed compact, near-spherical conformations of the analytes with scaling exponents of α = 0.44–0.54. These low α values indicate highly compact polymer structures caused by branching,^43^ in agreement with expectations from previous analyses. Although branching further limits the reliability of both conventional and universal calibration approaches,^44^ RI-based results were nevertheless employed to assess relative trends within the discussed sample series, owing to the broad availability and robustness of this detection method.

4 $$\left[\eta \right]=KM^{a}$$

where

- [η] = intrinsic viscosity/mL g^–1^
- *M* = molar mass/g mol^–1^
- *K* = system-specific constant/mL g^–1^ (g mol^–1^)^–a^
- *a* = scaling exponent (*a* = 0, hard sphere; *a* = 0.3–0.5, compact or branched polymers; *a* = 0.5–0.6, random coils under θ conditions)

As expected, a trend between functional group conversion and molecular weight could be observed, reaching *M*_n_ values of 5800–10 000 g mol^–1^ (SEC with pullulan calibration). One experiment (PES-06) was stopped right at the beginning of visible gelation. While the polyester was still water-soluble, light scattering gave a significant *M*_n_ of 5200 g mol^–1^. The hydrodynamic radius *R*_h,v_ (4.4 nm compared to 1.5–1.6 nm for other samples) indicated the formation of a nanogel that cannot be separated well due to the column exclusion limit. This result demonstrates that careful optimization of reaction parameters for individual synthesis apparatuses leads to CA-DMPA polyesters with significant chain lengths.

### 3.2 Synthesis of Neutralized Polyesters

Functionalization of CA-DMPA precondensates via postsynthetical neutralization of free carboxylic acid moieties with a strong base enhances the hydrophilicity of the polyester system. Due to the harsh pH conditions present, the neutralization step is linked to a reduction in molar mass,^7,9^ which is why the procedure was optimized in several regards (Table 2). The extent of hydrolysis hereby was examined via SEC (selected absolute molecular weights are given in the SI, Table S1). Contrary to polyester precondensates, for neutralized polyesters NMR analysis was not suitable due to an upfield shift of signals with increasing neutralization degree and thus increasing pH value. Exemplary spectra are shown in the SI (Figure S5). Further, neutralized polyesters did not display a *T*_g_ value anymore, as was determined with DSC.

**Table 2:** Summary of the Performed Neutralization Experiments,^a^ with Results Listed in the Form of Experimental Neutralization Degree (Based on Titration) and *M*_n_ Values (Based on Aqueous SEC-RI)

Sample	Solvent	*c*/wt %	Degree of neutralization (th.)/%	Degree of neutralization (exp.)/%	*M*_n_/g mol^–1^
PES-01_K75A	-	-	75	60	3200
PES-01_K75B	acetone	39	75	63	3400
PES-01_K75C	water	33	75	72	4500
PES-01_K75D	water	20	75	74	4600
PES-03_K25	water	20	25	26	6600
PES-03_K50	water	20	50	48	5800
PES-03_K75	water	20	75	71	5300
PES-03_K100	water	20	100	100	4000

a For the experimental series using PES-01 as precondensate, the reaction conditions were varied, while for the series using PES-03, only the theoretical degree of neutralization was changed.

The influence of solvent was investigated within experiments PES-01_K75A (no solvent), PES-01_K75B (acetone), and PES-01_K75C (water), targeting a neutralization degree of 75% with potassium hydroxide (“K75”). For PES-01_K75A, the 4 M KOH solution was added directly to the precondensate, leading to significant hydrolysis with the *M*_n_ value decreasing from 5900 g mol^–1^ (PES-01) to 3200 g mol^–1^ (conventional calibration). Hydrolysis was also reflected in the experimental degree of neutralization of 60%, which was lower than expected (75%) due to newly generated carboxy end groups. Dissolving the precondensate in acetone prior to KOH addition (39 wt % solution; PES-01_K75B) only led to a minor improvement, yielding an *M*_n_ of 3400 g mol^–1^. Significantly better results were achieved using water (33 wt % solution; PES-01_K75C), as seen by the *M*_n_ of 4500 g mol^–1^ and 72% neutralization degree being reached. Lastly, a different concentration of precondensate in water (20 wt % solution; PES-01_K75D) was tested, resulting in a neutralization degree of 74% and an *M*_n_ of 4600 g mol^–1^. Scale-up of the procedure by a factor of 10 was performed successfully. The experiments verified the correlation between harsh pH conditions and hydrolytic degradation of polyester chains.^7,9^

Tunability of the neutralization degree is highly desirable for many applications, as it enables engineering certain properties with regard to hydrophilicity and degradability while providing a certain number of free carboxylic acid groups for further modifications like cross-linking. A measurement series using PES-03 as precondensate (initial *M*_n_ of 7100 g mol^–1^) was conducted, where specific neutralization degrees of 25, 50, 75, and 100% could be synthesized successfully as verified by acid value measurements. To achieve higher neutralization degrees, more KOH is required, which in turn led to more drastic pH conditions and thus to more hydrolysis. Next to an increasing intensity of CA and DMPA peaks (Figure 3), an additional signal appeared around 8 min, which might be attributed to deprotonated citric acid species. This was indicated by SEC measurements of monosodium citrate, which showed a double peak for presumably both protonated and deprotonated species in the pH neutral NaNO_3_ solution. Calculated molar masses (conventional calibration) reflected the trend for hydrolysis well, where 100% neutralization led to a drastic decrease to an *M*_n_ of 4000 g mol^–1^. Contrarily, for 25% neutralization, larger molar masses of 6600 g mol^–1^ were retained.

**Figure 3:**
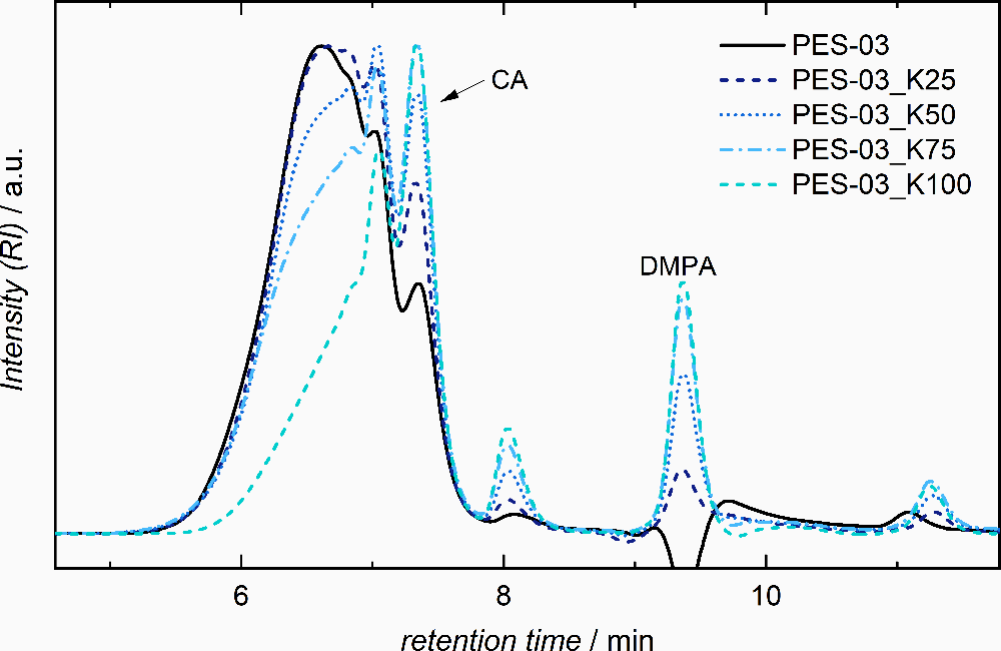
Size exclusion chromatograms of CA-DMPA precondensate PES-03 with varying neutralization degrees, including hydrolysis products (CA, citric acid; DMPA, dimethylolpropionic acid).

As an alternative to postsynthetical neutralization, synthesis with monosodium citrate (NaCA) was tested in combination with a selection of diol components. The utilization of an already functionalized monomer would simplify the overall synthesis pathway and reduce gelation due to fewer free carboxylic acid moieties. During synthesis with DMPA, the dominating reaction proved to be DMPA self-condensation, indicating insufficient reactivity of NaCA. MALDI measurements confirmed that only a minor amount of NaCA was incorporated into the polyester chains. Other diol components were tested as well, and no reasonable molar masses could be achieved. These results are contradictory to information found in the literature, where the synthesis of functional polyesters using NaCA was described as successful.^17^ Overall, it can be concluded that NaCA is not reactive enough for the intended esterification pathway and that significantly better results are achievable via the postsynthetical processing route.

### 3.3 Synthesis with Maleic Anhydride

Next to carboxylate groups, other charged moieties lead to the desired hydrophilicity, as well. An example would be sulfonate groups,^7,19,22^ where these are even reported to show superior thermal stability^15^ and hydrophilicity.^20^ Sulfonation was performed via the Michael addition of sulfite ions to conjugated double bonds.^19^ To incorporate the required unsaturated positions, maleic anhydride was used as a comonomer during synthesis, where incorporation takes place by means of a ring opening mechanism followed by a condensation reaction.^26^

The overall synthesis procedure was kept identical to that of two-component polyesters, with a melt polycondensation being conducted without a catalyst or an entrainer. The molar ratio between primary carboxy (CA and MAn) and hydroxy groups (DMPA) was again set at 1.05:1, where different ratios between MAn and CA were tested (Table 3). As was the case for two-component polyesters, the reaction mixture was prone to gelation. Apart from cross-linking via the available free carboxy groups, free hydroxy groups can also undergo conjugated addition to unsaturated positions as described by the Ordelt reaction.^45^ Due to an overlap of the corresponding NMR signals (2.4–2.9 ppm)^4^ with the CA region, it could not be determined to which extent the Ordelt reaction took place.

**Table 3:** Synthesis of CA-DMPA Copolyesters with Different Theoretical (Th.) and Experimental (Exp.) Molar Fractions (*x*) of Maleic Anhydride, with the Amount of COOH Groups and Double Bonds (DB) per Gram of Polyester as well as Estimated *M*_n_ Values (Based on THF-SEC, Polystyrene Calibration) Given

Sample	*x*_th._ MAn:CA	*x*_exp._ MAn:CA	*n*_COOH,th._/mmol g^–1^	*n*_DB,th._/mmol g^–1^	*n*_DB,exp._/mmol g^–1^	*M*_n_ (THF)^a^/g mol^–1^
PES-07	1:3	1:6.3	6.86	0.95	0.45	550
PES-08		1:3.6			0.81	610
PES-09	1:1	1:1.2	6.49	2.10	1.70	660
PES-10		1:1.3			1.63	760
PES-11	3:1	3:1.3	6.04	3.49	2.72	810
PES-12		3:1.4			2.57	920
PES-13	^b^	-	5.47	5.23	3.86	1230

a Values significantly underestimated in THF-SEC.

b MAn-DMPA homopolyester.

As indicated in Table 3, polyesters with different ratios between MAn and CA were synthesized to find a balance between the number of free carboxylic acid groups and double bonds (DB) within the polyester. Being able to tune this ratio allows one to synthesize polyesters with different properties. While the incorporation of CA leads to more free carboxylic acid groups that could be utilized for cross-linking, more MAn increases the amount of sulfonate groups and thus the hydrophilicity of the functionalized polyester. When comparing experimental values to the theoretical counterparts, differences in the amount of incorporated MAn became apparent, caused by sublimation^46^ and the subsequent removal of MAn via the applied negative pressure. This effect was especially drastic for PES-07, as the pressure was lowered immediately after the addition of DMPA to the reactor, leading to different molar ratios and a diol surplus. Lowering the pressure only after 1 h of reaction time at atmospheric pressure led to an improvement for synthesis with low MAn content (PES-08). An MAn loss of approximately 20–25% was observed for all syntheses, limiting chain growth and molar masses.

Due to decreasing solubility in water, only samples with a theoretical MAn content of maximum 50% could be measured in the aqueous SEC system. Molar masses were thus determined as polystyrene equivalents in THF, leading to a significant underestimation due to adsorption effects or varying conformations in the solvent as discussed in more detail in the SI (Section S2, Figure S12). For PES-09, *M*_n_/*M*_w_ values of 2000/3600 g mol^–1^ were achieved according to aqueous SEC using light scattering detection, while THF-SEC with RI detection and polystyrene calibration resulted in only 660/1130 g mol^–1^. Higher MAn ratios gave higher molar masses (Table 3), although adsorption and conformation effects might be dominating. While incomplete maleic anhydride incorporation changes the stoichiometric ratio of COOH and OH groups, leading to smaller chain lengths in linear systems, hydroxyl groups could still react with tertiary CA or DMPA carboxylic acid groups, promoting cross-linking and an increase of molecular mass. The *T*_g_ values were found to be between 28 and 52 °C. No clear dependence on maleic anhydride content could be observed, which might be explained by varying molar masses.

A characteristic region for MAn-containing polyesters within ^1^H NMR is the double bond region at approximately 6–7 ppm. Due to acid-catalyzed isomerization reactions, MAn can be present in the polyester chain as a maleic (*cis*) or fumaric (*trans*) skeleton.^26^ This is why signals within the double bond region can be assigned correspondingly,^42,47^ as shown in Figure S6. Assignment was performed with the help of ^13^C and 2D NMR (Figure S7). The DMPA methyl signals were not suitable to determine reliable OH conversion values of MAn-containing polyesters, contrary to the CA-DMPA systems. This is due to the significant downfield shift of signals with increasing MAn content, as reactions of DMPA with MAn lead to signals located more downfield than those corresponding to reactions with CA. Hereby, a more drastic shift is experienced for the fumaric acid isomer than for the maleic acid isomer.^48^ Due to this behavior, OH conversion would be overestimated as the DMPA-MAn monoester signals shift into the CA-DMPA diester region. The effect is visualized in Figure S8 (SI).

### 3.4 Sulfonation of Unsaturated Polyesters

The conjugated double bonds of maleic anhydride within the polyester chain can be functionalized via sulfonation with sulfite ions in aqueous solution, thus incorporating charged moieties. The newly attached sulfonate group led to a characteristic signal pattern in ^1^H NMR (Figure 4), as has been verified by comparison with the spectrum of sodium sulfosuccinate. Additionally, the signal density within the double bond region decreases significantly for sulfopolyesters because unsaturated positions are consumed during the sulfonation reaction. The successful incorporation of sulfonate groups was further confirmed by MALDI MS measurements (Figure 5). Full MALDI mass spectra can be found in the SI (Figure S14).

**Figure 4:**
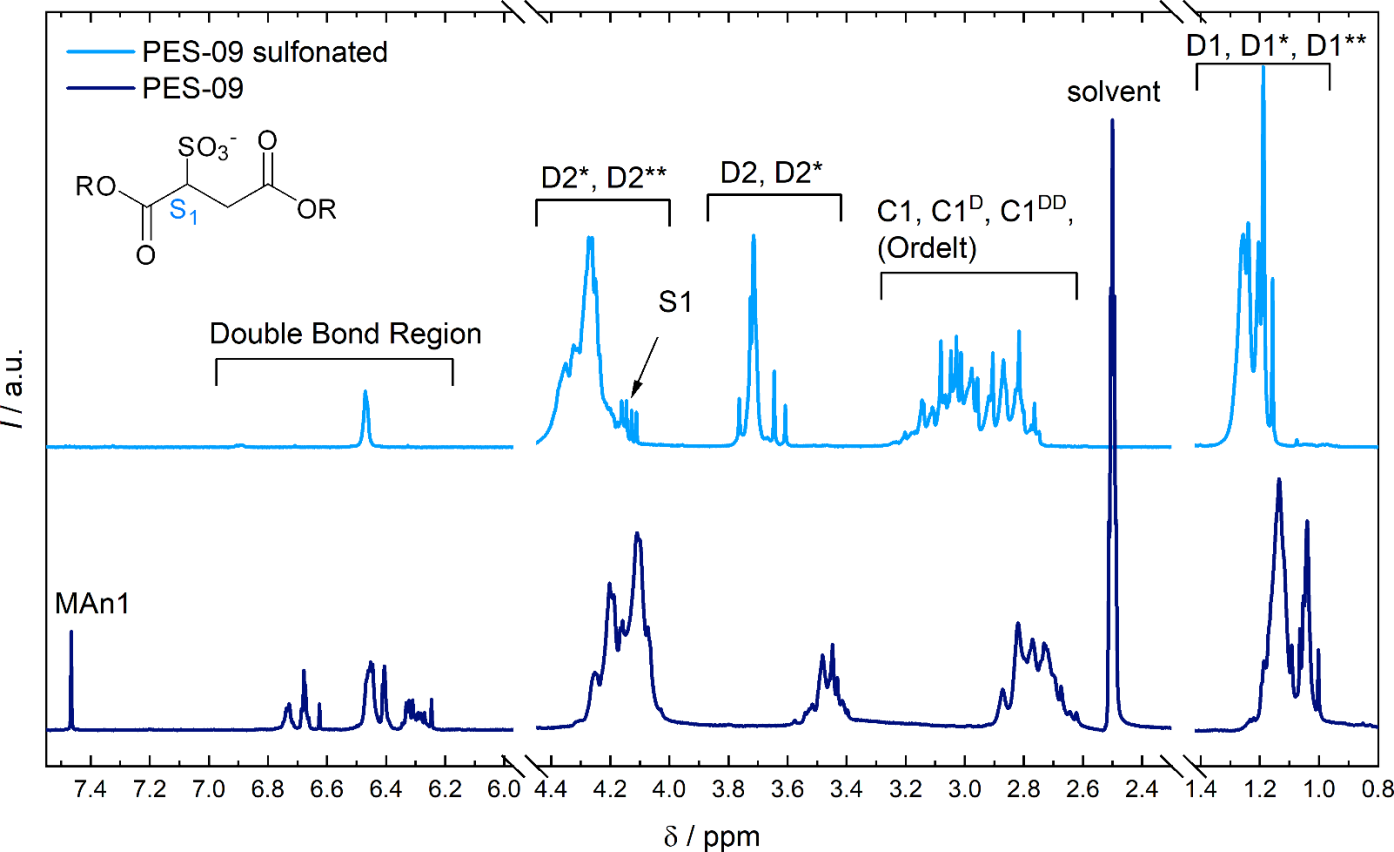
^1^H NMR spectra of the polyester sample PES-09 before (DMSO-*d*_6_) and after sulfonation (D_2_O). The signal S1 corresponds to the sulfonated position.

**Figure 5:**
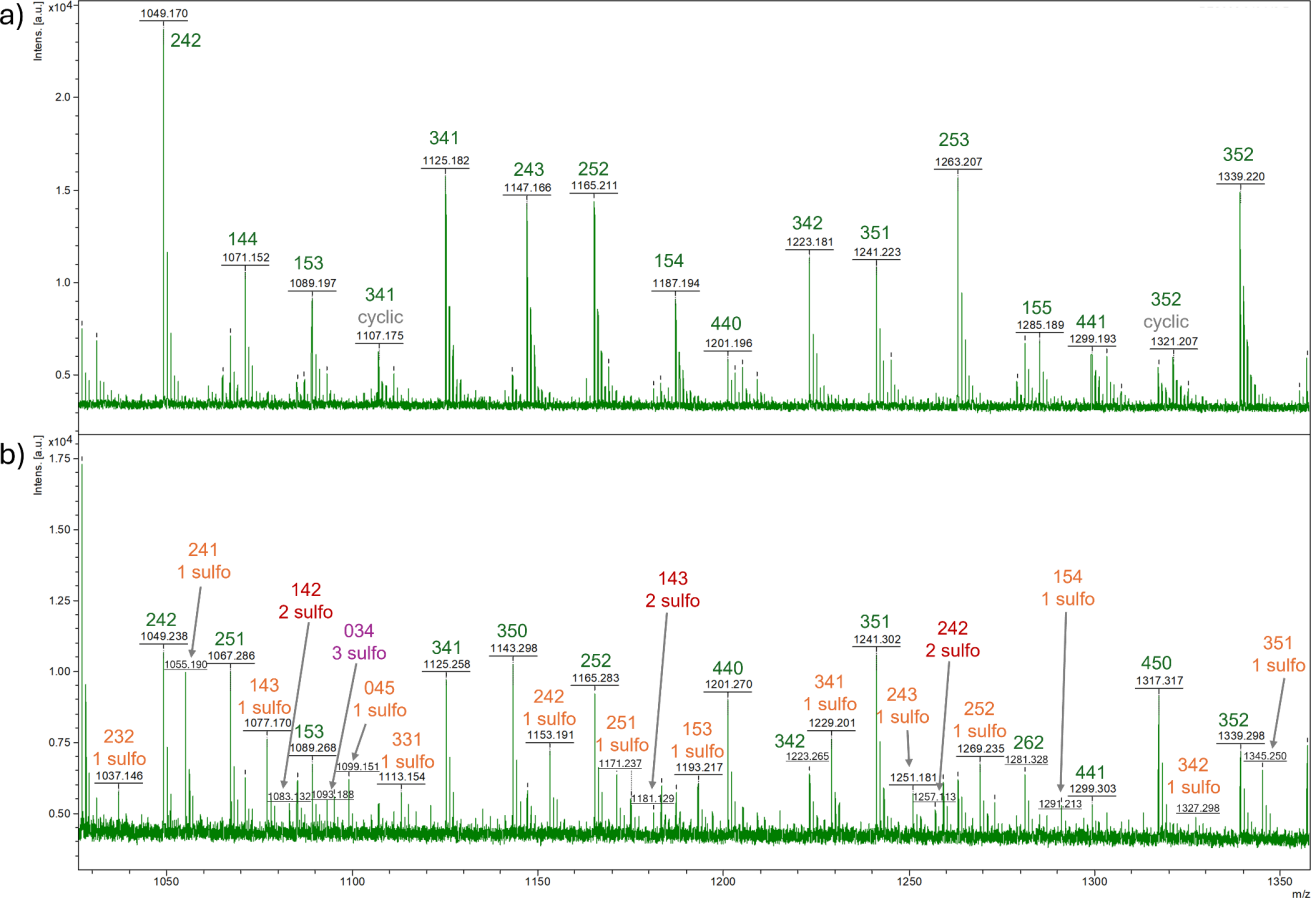
(a) MALDI-TOF mass spectrum of an unsaturated polyester (PES-09) containing CA, DMPA, and MAn and (b) the spectrum of a corresponding sulfopolyester (60 °C, 29 wt %). The peaks are labeled with their components: the first number corresponds to CA, the second number to DMPA, and the third number to MAn monomers. As all molecules are detected as Na adducts, they are not additionally indicated. Sulfonated molecules are labeled in either orange, red, or purple (depending on the amount of sulfonate groups added).

For optimization of the postsynthetical sulfonation procedure, several reaction parameters were screened. Clear trends were found for the performed temperature series (Figure 6a): sulfonation efficiency improved with increasing temperature, reaching 77 and 80% for 60 and 70 °C, respectively. The behavior is associated with the increased molecular motion and better availability of activation energy at higher temperatures as described by the Arrhenius law. The synthesis in aqueous solution additionally led to hydrolysis of ester bonds and thus a significant decrease of molar masses compared to the original precondensate. The rate of hydrolysis again depended on the temperature, reaching a drop of hydroxyl conversion from 83% for PES-09 to 69% for PES-09_S70 sulfonated at a reaction temperature of 70 °C. The decreasing molar mass was additionally confirmed via SEC (SI, Table S3). To find a balance between the opposing trends, a temperature of 60 °C was defined as a compromise, as high sulfonation degrees (77% ± 2.1% for 4 reactions) were achieved and acceptable OH conversions (74% ± 0.4%) retained. In case larger molar masses are required for subsequent applications of sulfopolyesters, cross-linking with the available free carboxy groups remains an attractive option.

**Figure 6:**
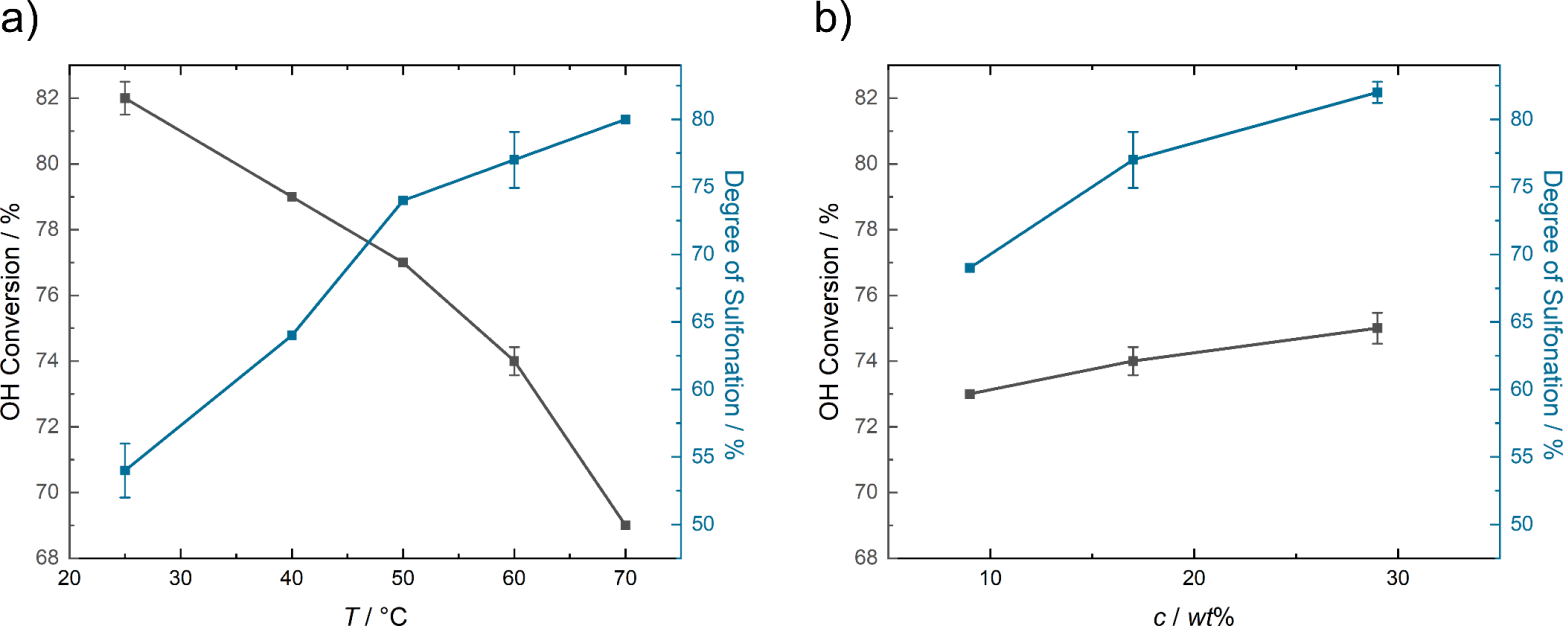
Degree of sulfonation and OH conversion related to polyester hydrolysis for sulfonation experiments performed at (a) temperatures of 25–70 °C and (b) varying concentrations.

As a second parameter, the amount of solvent during sulfonation was varied (using a temperature of 60 °C), where 4 g of polyester was dissolved in 40, 20, and 10 mL of water, corresponding to concentrations of 9, 17, and 29 wt % respectively. Sulfonation efficiency (Figure 6b) improved with increasing concentration, reaching 82% ± 0.8% (3 reactions) at 29 wt %. At the same time, hydrolysis was reduced, leading to a slightly higher OH conversion of 75% ± 0.5% (3 reactions). Concerning the molar mass, SEC results indicated an improvement going from 9 wt % (*M*_n_ of 4600 g mol^–1^) to 17 wt % (*M*_n_ of 5000 g mol^–1^), while 17 and 29 wt % led to comparable results. Overall, smaller solvent volumes optimize the sulfonation process, not only due to increased sulfonation efficiency but also because fewer resources are consumed and less water needs to be removed.

The optimization of the sulfonation procedure was mostly based on the sulfonation degree calculated by quantitative ^1^H NMR using the double bond peak region. To determine whether side reactions such as hydration of double bonds via the Ordelt reaction significantly impact the perceived results, control experiments were conducted. The results indicated that side reactions could account on average for 2% of double bond reactions. Hydrolysis took place for the control experiments as well, verifying that not the sulfonation agent but the aqueous medium and increased temperature were responsible for the decrease in molar mass.

Sulfonation (*T* = 60 °C, *c* = 29 wt %) was tested for several unsaturated polyester precondensates (PES-08 to PES-13) with varying maleic anhydride content. The effect of precondensate composition on sulfonation efficiency was examined on both small (4 g) and large scales (40 g), and the experiments are listed in Table 4. The sulfonation efficiency increased with an increasing amount of maleic anhydride. While PES-08 (4 g) displayed only a 64% degree of sulfonation (0.56 mmol SO_3_^–^ g^–1^), 92% was achieved for PES-11 (4 g), which corresponds to 2.28 mmol of SO_3_^–^ per gram of polyester.

**Table 4:** Results of Sulfonation Experiments Performed on Different Scales and for Different Ratios between Maleic Anhydride and Citric Acid within the Polyester Precondensates^a^

Sample	Batch size^a^/g	*x*_th._ MAn:CA	OH conv. (NMR)/%	Degree of sulfonation/%	*n*(SO_3_^–^)/mmol g^–1^
PES-08	4	1:3	74	64	0.56
PES-09	4	1:1	75 ± 0.5	82 ± 0.8	1.40
	40	1:1	75	85	1.45
PES-10	40	1:1	-^b^	79	1.36
PES-11	4	3:1	-^b^	92	2.28
	40	3:1	-^b^	91	2.26
PES-12	40	3:1	-^b^	91	2.26
PES-13	40	^c^	-^b^	78	2.68

a For PES-09 (4 g), average values are shown, as several experiments were conducted under identical conditions.

b Mass of polyester precondensate used for sulfonation.

c No sufficient peak resolution in the NMR spectrum.

d MAn-DMPA homopolyester.

This trend is in accordance with the literature.^19^ An exception was the sulfonation of MAn-DMPA homopolyester (PES-13) which took significantly longer to be completely dissolved during the reaction, thus reducing the sulfonation efficiency.

Generally, an increasing number of sulfonated positions led to a significant broadening of the DMPA methyl signals within ^1^H NMR (SI, Figure S10). This is why a reasonable determination of OH conversion was not possible for all experiments. SEC-RI results as summarized in the SI (Table S1) showed a decrease of molar masses with a maximum of 1000 g mol^–1^, e.g., from 5600 to 4800 g mol^–1^ for PES-09 (40 g scale). The molar mass decrease of several samples could not be directly compared due to the solubility change of the unsaturated polyesters. Absolute molar masses derived from the light scattering detector gave an *M*_n_ of 2000 g mol^–1^ and *M*_w_ of 5100 g mol^–1^ for PES-11, and even higher chain lengths were reached for PES-12 considering the available SEC-RI results. The sulfopolyesters show a high concentration of functional groups (COOH, SO_3_^–^) and reasonable molar masses, which make them very promising precondensates for further research on cross-linking and water-absorbing materials.

## 4 Conclusion

Two approaches are presented for the synthesis of water-soluble precondensates containing both charged functionalities for high hydrophilicity and carboxylic acid groups for succeeding cross-linking to develop sustainable materials with water-absorbing properties. First, polyesters are synthesized from biobased citric acid and dimethylolpropionic acid via uncatalyzed melt polycondensation and further modified by neutralization with varying amounts of potassium hydroxide. Second, the synthesis of unsaturated polyesters using maleic anhydride as a third monomer provides conjugated double bonds available for the addition reaction of sodium sulfite to introduce sulfonate groups. The termination of the polycondensation reactions proves to be crucial to obtain reasonable chain lengths but avoid gelation, which is primarily caused by reactions of tertiary carboxylic acid groups and potential diol addition to unsaturated building blocks. Since neutralization and sulfonation reactions are performed in water, polyesters are partially hydrolyzed, especially under the basic conditions present during KOH addition for neutralization. Optimization of reaction parameters limited hydrolysis, especially for sulfopolyesters, while molar masses of neutralized CA-DMPA polyesters strongly depend on the degree of neutralization. All of the described precondensates contain a high number of functional groups (−COOH, −COO^–^, −SO_3_^–^) with varying compositions and carry the potential to obtain sustainable materials with tunable absorption and degradation properties after cross-linking.

## Supplementary Material



## Data Availability

Raw and additional processed data will be provided upon request.

## Notes

^1^H and ^13^C NMR spectra of CA-DMPA­(-MAn) polyester precondensates; ^1^H NMR spectra of functionalized (neutralized/sulfonated) polyesters; details concerning SEC measurements and HPLC-MS measurements; full MALDI spectra; and details concerning the analysis of sulfopolyesters (PDF)
