# Supplementary material for: Synthesis of Functional Water-Soluble Polyesters Based on Citric Acid and Dimethylolpropionic Acid
Source: ACS Polym Au. 2026 Feb 20;6(2):610–21. doi: 10.1021/acspolymersau.5c00196 (PMC13067171; doi:10.1021/acspolymersau.5c00196)
Supplement: Supplementary file 1 [file lg5c00196_si_001.pdf]

Synthesis of Functional Water-Soluble Polyesters Based on Citric Acid and Dimethylolpropionic Acid

Anna Kruglhuber[a], Clemens Bernhard[a], Susanne Boye[b], Markus Wierer[c], Albena Lederer[b,d], Clemens Schwarzinger[a,*], Klara M. Saller[a,*]

[a]Institute for Chemical Technology of Organic Materials, Johannes Kepler University Linz, Altenbergerstrasse 69, 4020 Linz, Austria

[b]Leibniz-Institut für Polymerforschung Dresden, Department Advanced Macromolecular Structure Analysis, Hohe Strasse 6, 01069 Dresden, Germany

[c]Institute for Analytical Chemistry, Johannes Kepler University Linz, Altenbergerstrasse 69, 4020 Linz, Austria

[d]Stellenbosch University, Department Chemistry and Polymer Science, Private Bag X1, Matieland 7602, South Africa

*Corresponding authors. E-mail addresses: klara.saller@jku.at, clemens.schwarzinger@jku.at, telephone: +43 732 2468 9000.

Table of Contents

1. NMR ... 2
   1.1. Synthesis with Citric Acid and Dimethylolpropionic Acid ... 2
   1.2. Synthesis of Neutralized Polyesters ... 4
   1.3. Synthesis with Maleic Anhydride ... 5
   1.4. Sulfonation of Unsaturated Polyesters ... 7
2. SEC ... 9
3. HPLC-MS ... 11
4. MALDI Mass Spectrometry ... 12
5. Analysis of Sulfopolyesters ... 13

1. NMR

1.1. Synthesis with Citric Acid and Dimethylolpropionic Acid

The OH conversion of synthesized polyesters (CA-DMPA polyesters) was calculated using the DMPA methyl signals in the ^{1}H NMR spectrum. The signal consists of three distinct regions, which can be assigned to diester (D1^{CC}), monoester (D1^{C}) and monomer (D1) respectively. The superscripts hereby indicate the reaction partner(s), with “C” standing for citric acid (Figure S1).

In Figure S2, the ^{13}C NMR of PES-01 (CA-DMPA polyester) is shown, peaks are assigned according to the presented nomenclature system. Reaction partner(s) are again indicated *via* superscripts, with “C” and “D” standing for citric acid and dimethylolpropionic acid respectively.

Peak assignments were performed with and/or verified with 2D NMR measurements. The corresponding HSQC and HMBC correlation spectra are shown below (Figures S3 and S4), whereby peaks are labelled according to the introduced nomenclature system.

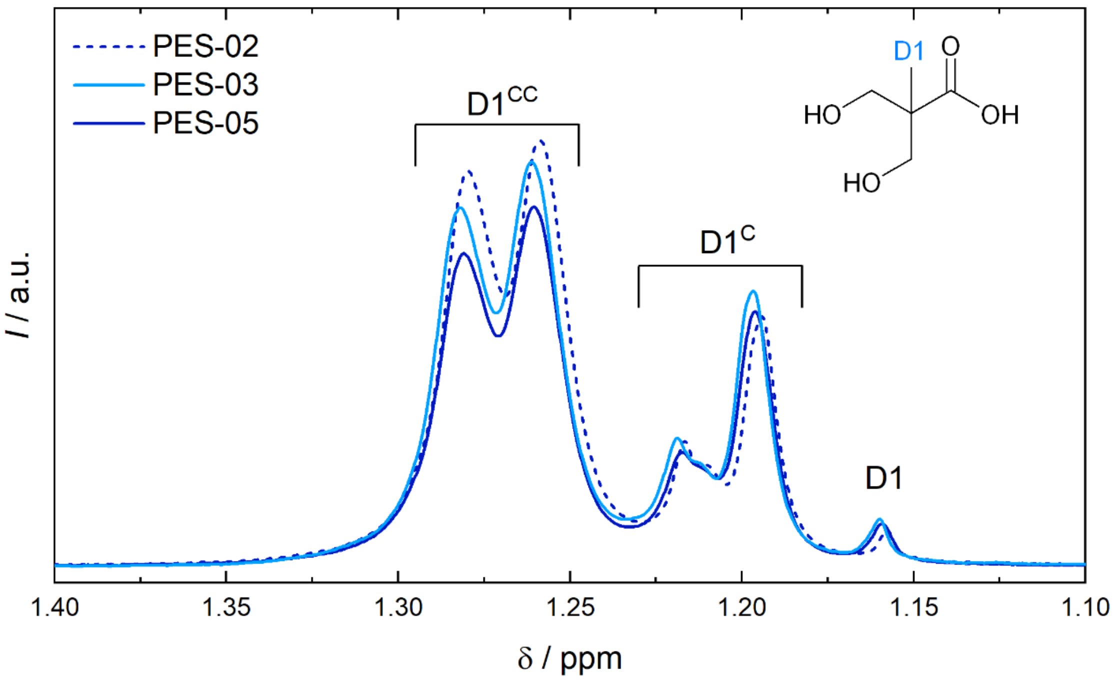

Figure S1: ^{1}H NMR of PES-02, PES-03 and PES-05 (in D_2O). Illustrated is the NMR region ($-CH_3$ signal of DMPA) that was used for calculation of the OH conversion. Peaks are assigned to DMPA diester (D1^{CC}), DMPA monoester (D1^{C}), or DMPA monomer (D1), whereby the superscript “C” refers to citric acid being the reaction partner.

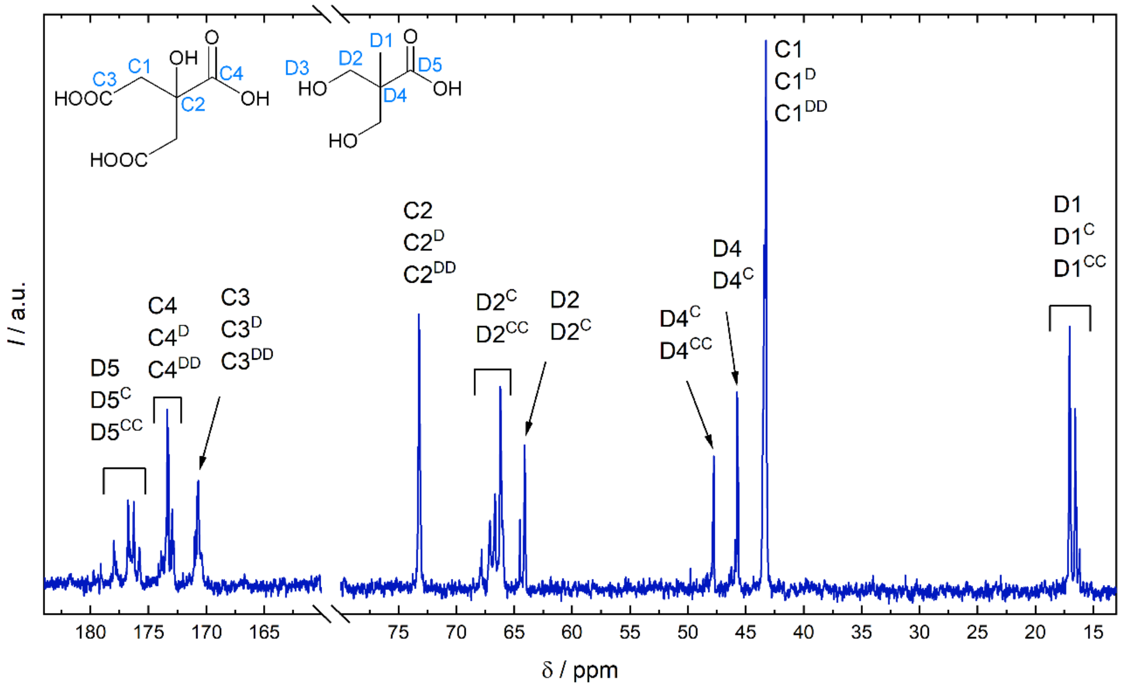

Figure S2: The ^{13}C NMR of PES-01 (CA-DMPA polyester) is shown. The depicted structures show the nomenclature system according to which all signals are labelled. Superscripts indicate the reaction partner(s), whereby “C” stands for citric acid, and “D” for dimethylolpropionic acid.

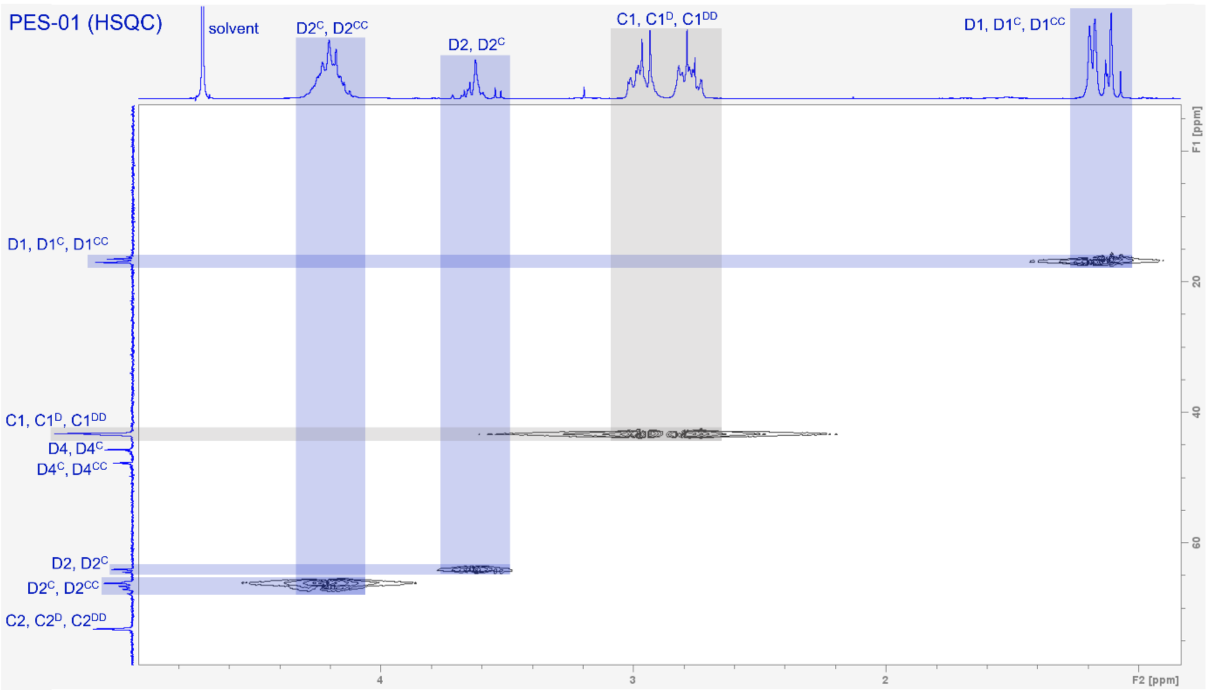

Figure S3: HSQC correlation spectrum of PES-01 (CA-DMPA polyester).

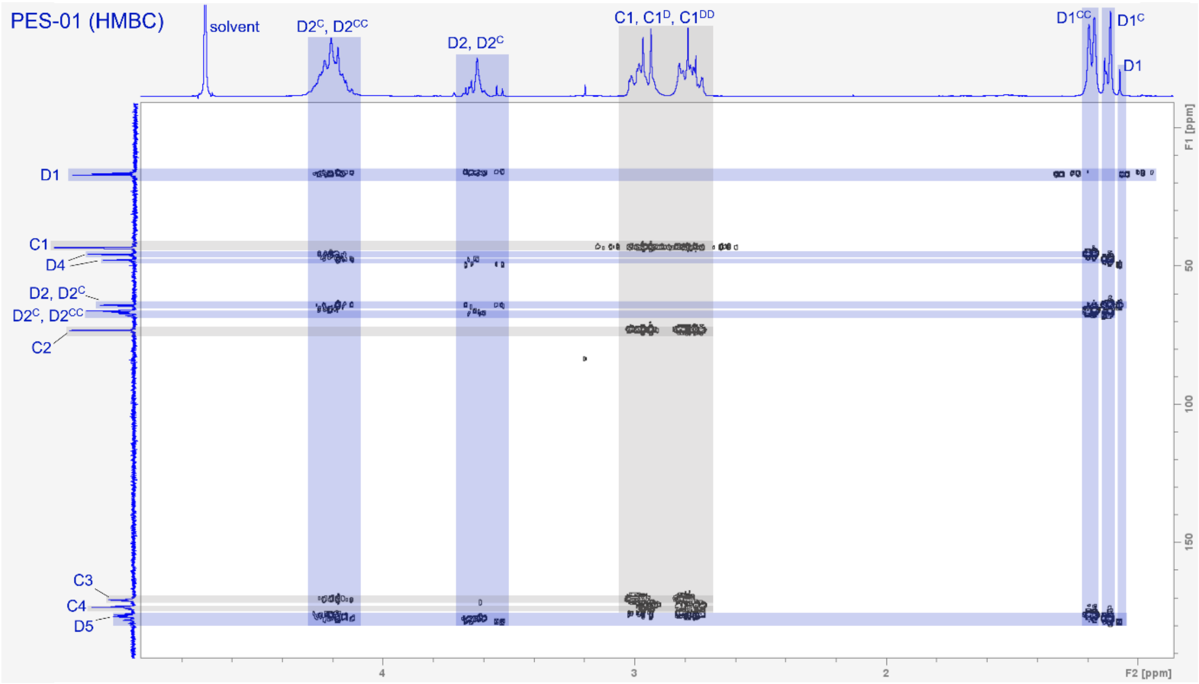

Figure S4: HMBC correlation spectrum of PES-01 (CA-DMPA polyester).

1.2. Synthesis of Neutralized Polyesters

Post-synthetical neutralization of CA-DMPA polyester precondensates was performed, whereby different neutralization degrees were engineered. With an increasing degree of neutralization an upfield shift of signals can be observed, especially for the $C1/C1^{D}/C1^{DD}$ and $D1/D1^{C}/D1^{CC}$ signal regions (Figure S5).

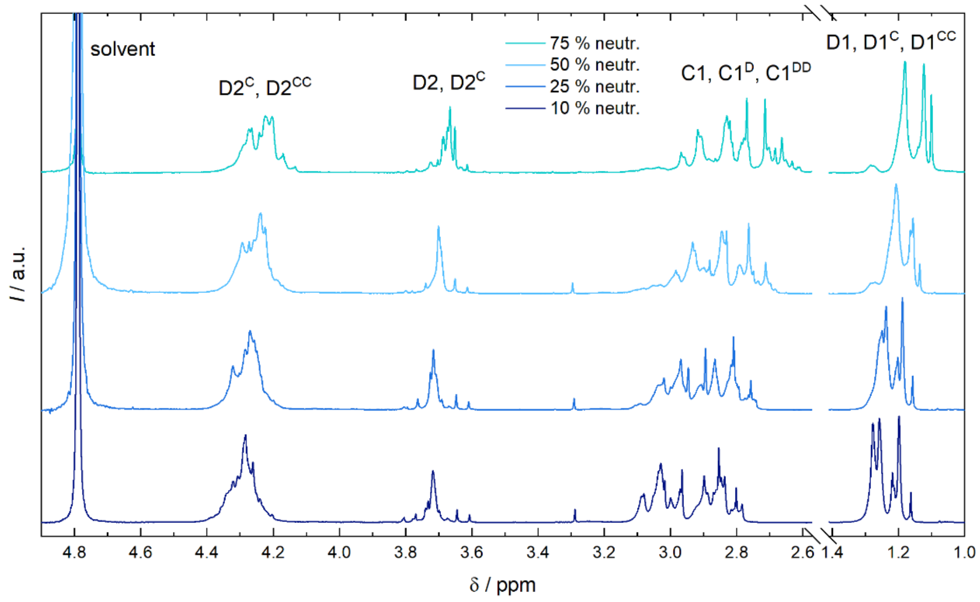

Figure S5: Different neutralization degrees (10, 25, 50, and 75 %) were engineered for PES-01 (in D_2O). Corresponding ^{1}H NMR measurements are depicted, whereby an upfield shift can be observed for C1/C1^D/C1^{DD} and D1/D1^C/D1^{CC} regions.

1.3. Synthesis with Maleic Anhydride

Three-component polyesters were synthesized using maleic anhydride as an additional monomer, leading to incorporation of unsaturated positions. The double bond region of ^{1}H NMR displays a specific signal pattern because maleic anhydride is incorporated either as a maleic acid ("M") or a fumaric acid skeleton ("F"), as can be seen in Figure S6. The signals were assigned with the help of 2D NMR, whereby the corresponding ^{13}C NMR is shown in Figure S7. Reaction partner(s) are indicated in the superscripts ("N" standing for neopentyl glycol and "D" standing for DMPA), with different reaction partners leading to slight overall shift of signals. An asterisk as superscript indicates an unassigned reaction partner.

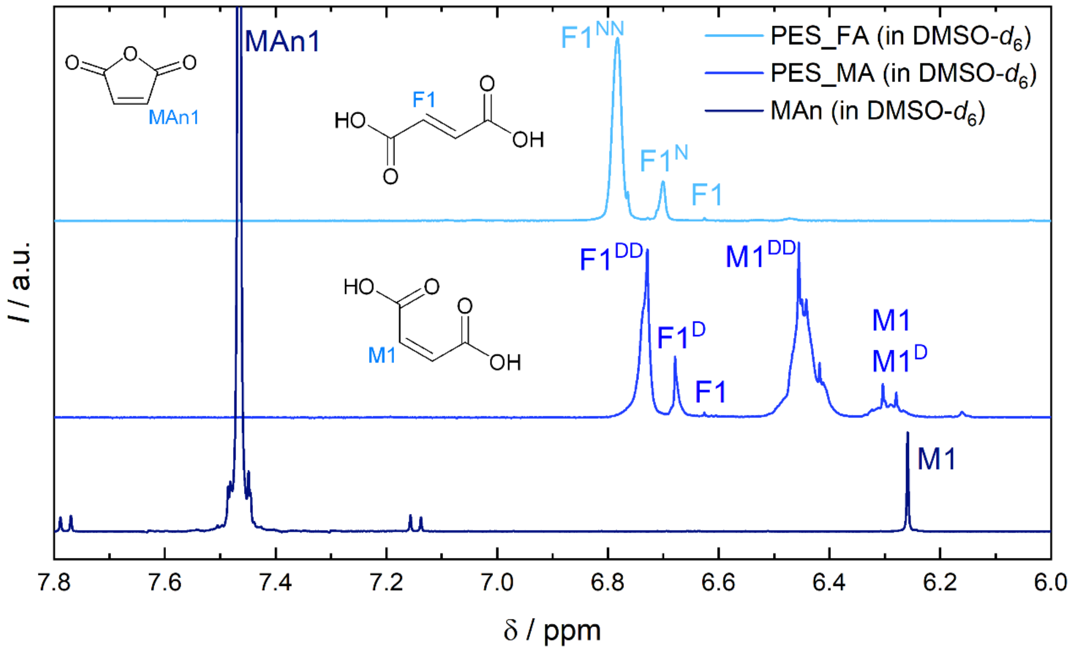

Figure S6: The double bond region of unsaturated polyesters (^{1}H NMR measurements in DMSO-d_6) is shown in more detail. Depicted are spectra of maleic anhydride (bottom curve), a polyester containing both fumaric and maleic skeletons (middle curve), and a polyester containing only fumaric skeletons (top curve). Reaction partner(s) are indicated by the superscripts, whereby "D" stands for DMPA, and "N" for neopentyl glycol.

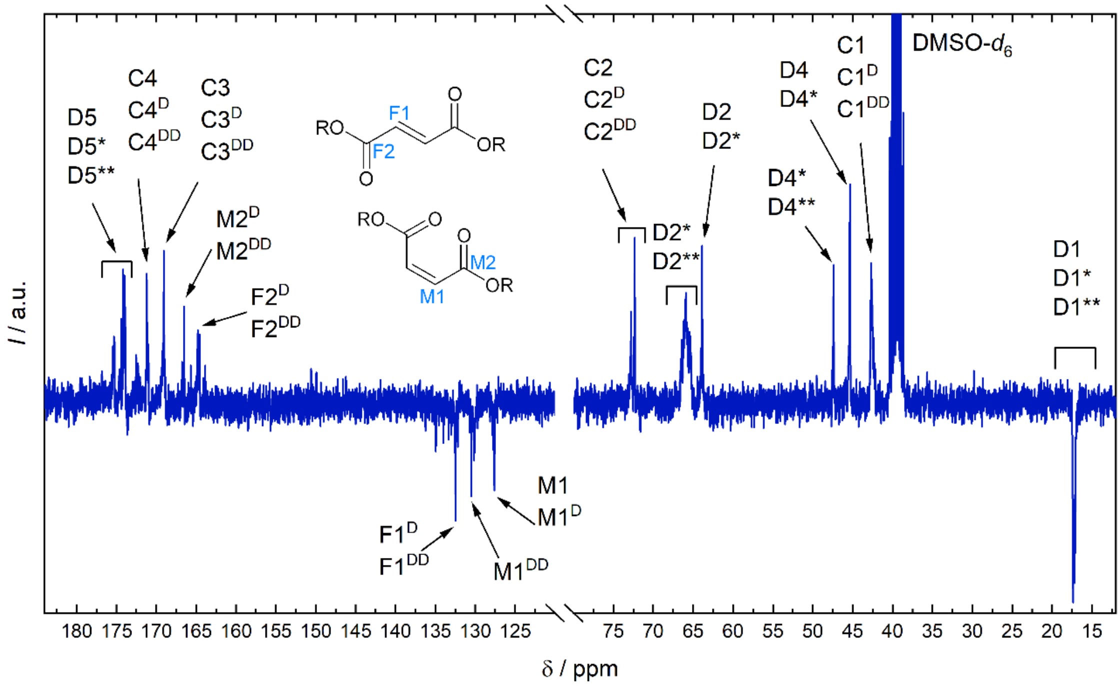

Figure S7: The ^{13}C-APT NMR of PES-09 is shown. Signals are labelled according to the introduced nomenclature system. Maleic anhydride is either incorporated as its maleic ("M") or fumaric ("F") skeleton, whereby both isomers lead to specific signals that are labelled according to the depicted structures. Superscripts indicate the reaction partner(s), whereby "D" stands for DMPA, and an asterisk stands for an unassigned reaction partner.

With an increasing amount of maleic anhydride, a significant broadening of the DMPA methyl signal in ^{1}H NMR can be observed (Figure S8). This is because reactions of DMPA with MAn lead to signals located more downfield than those corresponding to reactions with CA. Further, the shift is more drastic for the fumaric acid isomer than for the maleic acid isomer.

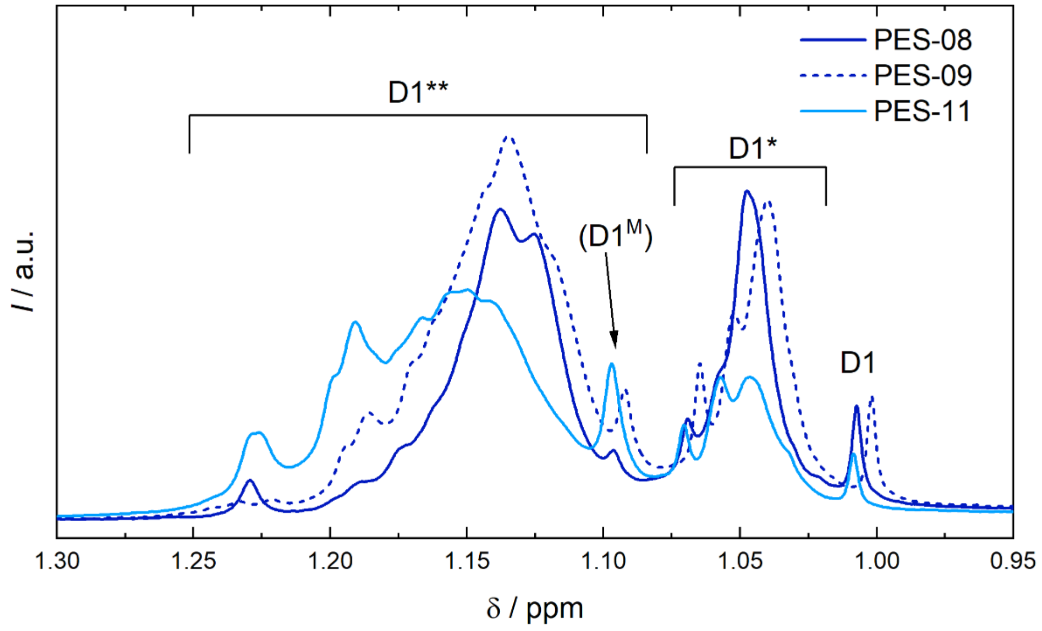

Figure S8: The ^{1}H NMR methyl region of DMPA (in DMSO-d_6) is shown for three polyesters containing different amounts of MAn (PES-08 < PES-09 < PES-11). Regions are assigned to DMPA monomer (D1), DMPA monoester (D1*) and diester (D1**), whereby the asterisks indicate that no specific reaction partner was assigned. A downfield shift in signals can be observed for reaction of DMPA with MAn compared to reaction with CA. The presumed monoester signal of DMPA with MAn incorporated as a maleic acid skeleton (D1^M) is shown.

1.4. Sulfonation of Unsaturated Polyesters

For determination of the degree of sulfonation quantitative ^{1}H NMR measurements were used. For polyester precondensates, measurements were conducted in DMSO-d_6 using benzoic acid as internal standard, while sulfopolyesters were measured in D_2O using potassium hydrogen phthalate as internal standard. Corresponding ^{1}H NMR regions are shown in Figure S9. With an increasing degree of sulfonation, a significant peak broadening can be observed for the DMPA methyl region in ^{1}H NMR (Figure S10), making determination of the OH conversion unreasonable for certain experiments.

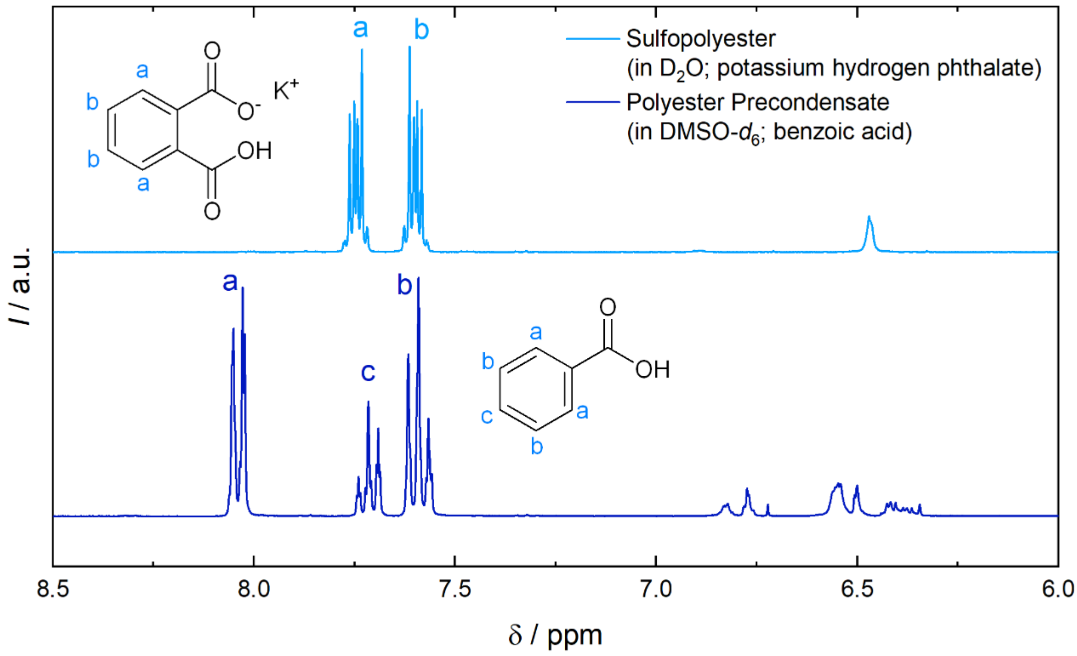

Figure S9: Representative ^{1}H NMR spectra illustrating how the amount of double bonds was determined with quantitative NMR measurements (using potassium hydrogen phthalate or benzoic acid as internal standards). The spectra of a sulfopolyester (in D_2O) as well as a polyester precondensate prior to sulfonation (in DMSO-d_6) are depicted. Signals within the double bond region (6.20–7.00 ppm) are less prominent for the sulfopolyester, indicating a successful sulfonation reaction.

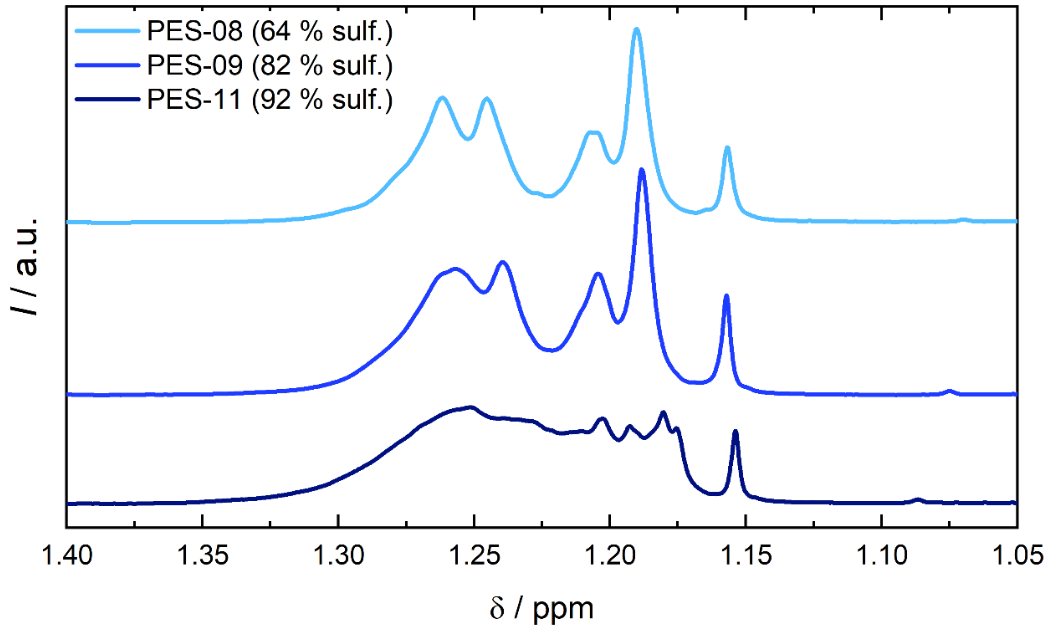

Figure S10: The ^{1}H NMR spectra (DMPA methyl region; in D_2O) of three sulfonated polyesters (small scale experiments) are shown. With increasing sulfonation degree (PES-08 < PES-09 < PES-11) a significant broadening of peaks can be observed, which makes integration and thus determination of the OH conversion unreasonable for some experiments.

2. SEC

SEC analysis was performed on two different set-ups using either an aqueous $NaNO_3$ buffer or THF as solvent. Results were strongly deviating as summarized in Table S1. Limitations of the aqueous SEC using refractive index detection is discussed in the main text (Section 3.1.). Most accurate results for branched polyesters were obtained by light scattering detection with fitting (Figure S11) to compensate low signal intensities for small molecular weight fractions.

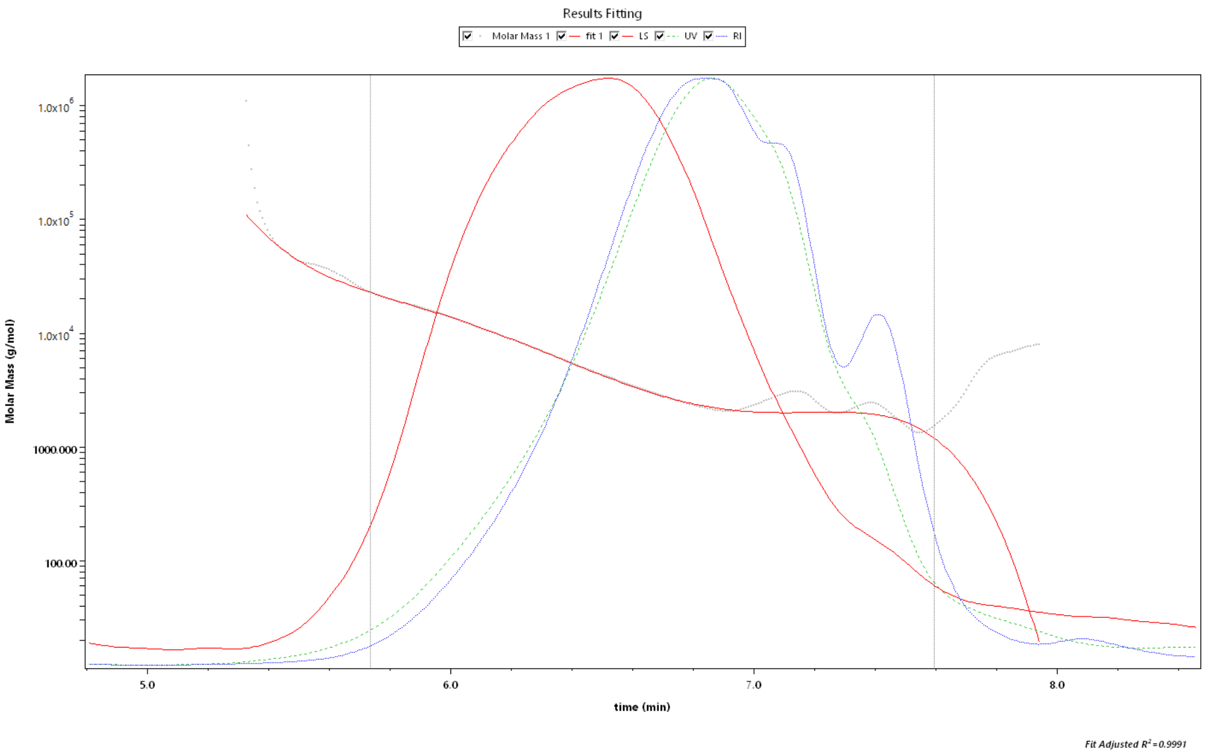

Figure S11: Fitting of the light scattering signal eliminated mass inaccuracies especially in the lower molecular weight region.

Using THF as eluent overlapping of analyte signals with negative peaks of the refractive index detector was observed. This is likely due to strong adsorption effects and/or varying conformation of the analytes in the solvent which seemed to decrease with increasing maleic anhydride content (Figure S12). If possible, results from the aqueous SEC were preferred for direct comparison with water-soluble (and THF-insoluble) charged products after neutralization or sulfonation. For polyesters with significant maleic anhydride contents (75 % MAn, theoretical), water solubility was lost and molar masses were estimated using the THF-SEC and polystyrene calibration.

Table S1: Number and weight average molecular masses (M_n, M_w / g mol^{-1}) derived from different SEC methods (RI…refractive index, MALS…multi-angle light scattering). Aqueous SEC was calibrated with pullulan standards, polystyrene was used for the calibration of the THF SEC.

	**aqueous SEC**					**THF SEC**	
	RI		LS			RI	
Sample	M_n	M_w	M_n	M_w	dn/dc	M_n	M_w
PES-01	5900	9200	3400	5300	0.124	360	530
PES-02	7600	15100					
PES-03	7100	12700	2100	4000	0.131	410	580
PES-04	5800	8900					
PES-05	6700	11900					
PES-06	10000	49600	5200	50800	0.131	540	950
PES-01_K75A	3200	6200					
PES-01_K75B	3400	6800					
PES-01_K75C	4500	7400					
PES-01_K75D	4600	7400					
PES-03_K25	6600	11900	2100	4100	0.132		
PES-03_K50	5800	11300	1900	3800	0.134		
PES-03_K75	5300	11100	1800	3600	0.135		
PES-03_K100	4000	7400	1300	2600	0.137		
PES-07						550	930
PES-08	5900	10400	1800	2900	0.136	610	1060
PES-09	5600	10100	2000	3600	0.136	660	1130
PES-10						760	1600
PES-11						810	1750
PES-12						920	2340
PES-13						1230	5990
PES-09_S25	5600	10500					
PES-09_S40	5400	9600					
PES-09_S50	5300	9000					
PES-09_S60	5000	8000					
PES-09_S70	4300	6700					
PES-09_S09%	4600	7600					
PES-09_S29%	4900	7900					
PES-08_SU	4900	8100	1900	2600	0.133		
PES-09_SU	4800	7900	1700	2400	0.133		
PES-10_SU	5500	12900					
PES-11_SU	5600	11500	2000	5100	0.133		
PES-12_SU	6200	17900					
PES-13_SU	5200	21500	3300	22400	0.133		

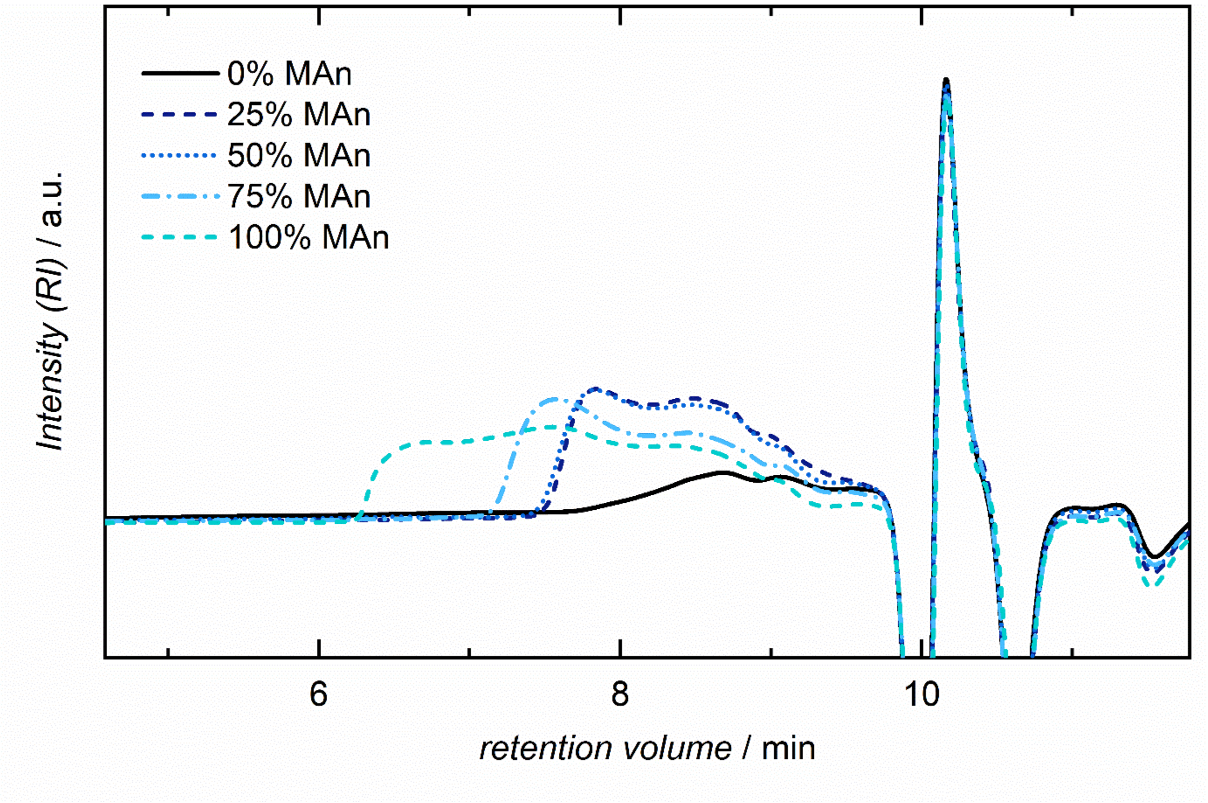

Figure S12: THF chromatograms for polyesters with varying maleic anhydride content (0 %...PES-03, 25 %...PES-08, 50 %...PES-09, 75 %...PES-11, 100 %...PES-13).

3. HPLC-MS

HPLC-MS measurements were conducted. Details concerning the utilized water/acetonitrile gradient can be found in Table S2. Measurement results indicated, that the reactivity of the tertiary hydroxyl group of citric acid can be neglected (Figure S13).

Table S2: Gradient used for HPLC-MS measurements performed at a flow rate of 0.5 mL min^{-1} and a runtime of 30 minutes.

**Time / min**	**Water / % *v/v***	**Acetonitrile / % *v/v***
0	90	10
17	10	90
22	0	100
25.9	0	100
26	90	10

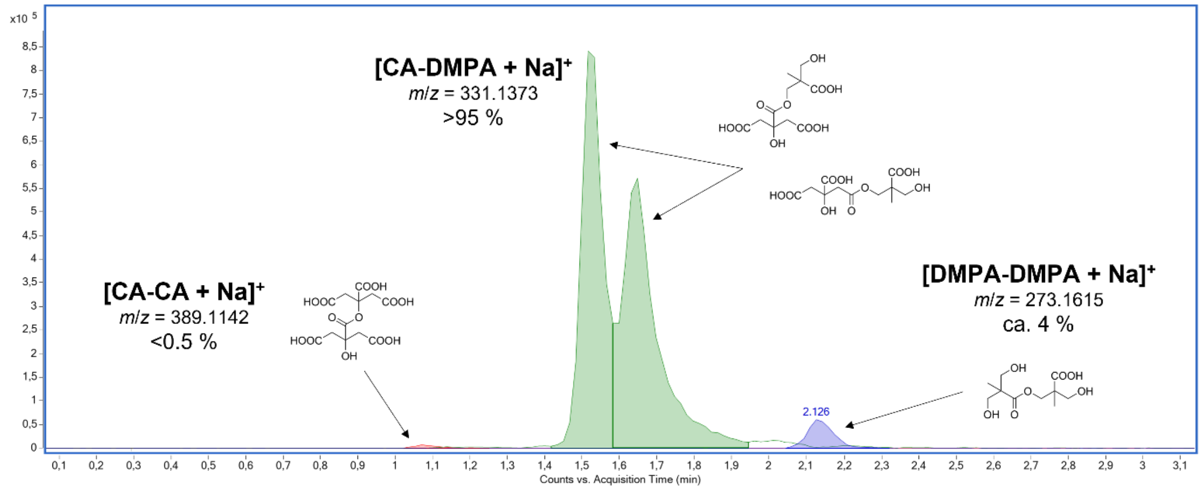

Figure S13: HPLC-MS measurements revealed that mostly citric acid (CA) and dimethylolpropionic acid (DMPA) reacted with each other while a small fraction of DMPA reacted with itself (DMPA-DMPA). Only traces of citric acid dimer could be found.

4. MALDI Mass Spectrometry

The full mass spectra of a selection of samples (PES-05, PES-09 and PES-09_S60_29%) in reflectron mode are shown in Figure S14. With the rising complexity of the polyester structure (PES-05: CA-DMPA, PES-09: CA/MAn-DMPA) the number of oligo- and polymeric species and their corresponding MALDI peaks increased. This led to less distinct peaks in higher m/z regions while SEC suggested higher molar masses for unsaturated polyesters. Sulfonation of the polyesters (PES-09_S60_29%) led to an even more complex MALDI spectrum. Additionally, sulfopolyesters seemed to have lower ionization efficiencies compared to polyester chains containing residual maleic acid units at sulfonation degrees of 90 % and higher. The spectrum of PES-03 recorded in linear mode was used for a molar mass calculation with the Bruker PolyTools 2.0 software (Figure S15).

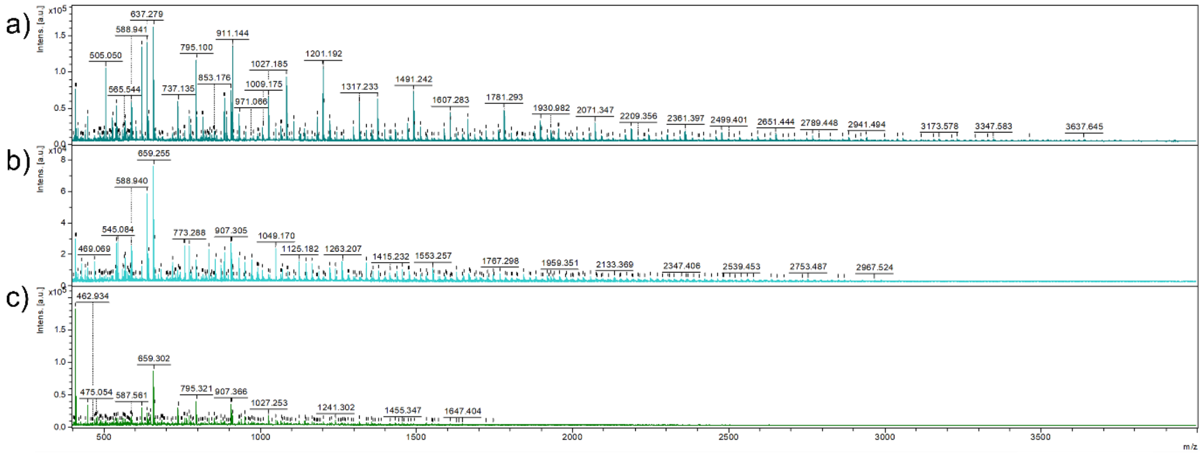

Figure S14: Full MALDI-TOF mass spectra of a) PES-05, b) PES-09 and c) PES-09_S60_29%. The spectra were recorded in reflectron mode.

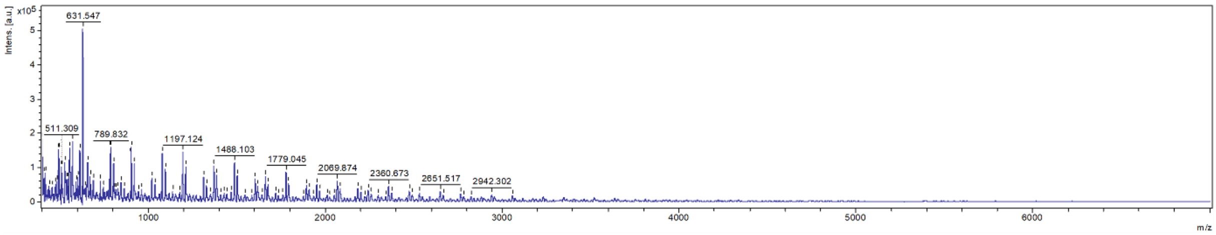

Figure S15: Full MALDI-TOF mass spectrum of PES-03. As this spectrum was used for molecular weight calculation, it was recorded in linear mode.

5. Analysis of Sulfopolyesters

The post-synthetical sulfonation procedure was optimized concerning reaction temperature and concentration. The corresponding results are listed in Tables S3 and S4.

Table S3: Selection of sulfonation experiments performed within the temperature screening experimental series, whereby temperatures between 25 and 70 °C were tested. If several repetitions were performed, the average values as well as the corresponding standard deviation are indicated. M_n and M_w values are based on SEC-RI measurements.

***T* / °C**	**OH Conversion (NMR) / %**	**Degree of sulfonation / %**	**M_n / g mol^{-1}**	**M_w / g mol^{-1}**
25	82 ± 0.5	54 ± 2.0	5600	10500
40	79	65	5400	9600
50	77	74	5300	9000
60	74 ± 0.4	77 ± 2.1	5000	8000
70	69	80	4300	6700

Table S4: Overview of sulfonation experiments performed at different concentrations (9, 17 and 29 *wt*%). If several repetitions were performed, the average values as well as the corresponding standard deviation are indicated. M_n and M_w values are based on SEC-RI measurements.

***c* / *wt*%**	**OH Conversion (NMR) / %**	**Degree of sulfonation / %**	**M_n / g mol^{-1}**	**M_w / g mol^{-1}**
9	73	69	4600	7600
17	74 ± 0.4	77 ± 2.1	5000	8000
29	75 ± 0.5	82 ± 0.8	4900	7900
